# Multiplexed CRISPR-Cas9 mutagenesis of rice *PSBS1* noncoding sequences for transgene-free overexpression

**DOI:** 10.1126/sciadv.adm7452

**Published:** 2024-06-07

**Authors:** Dhruv Patel-Tupper, Armen Kelikian, Anna Leipertz, Nina Maryn, Michelle Tjahjadi, Nicholas G. Karavolias, Myeong-Je Cho, Krishna K. Niyogi

**Affiliations:** ^1^ Department of Plant and Microbial Biology, University of California, Berkeley, CA 94720, USA.; ^2^ Howard Hughes Medical Institute, University of California, Berkeley, CA 94720, USA.; ^3^ Innovative Genomics Institute, University of California, Berkeley, CA 94720, USA.; ^4^ Molecular Biophysics and Integrated Bioimaging Division, Lawrence Berkeley National Laboratory, Berkeley, CA 94720, USA.

## Abstract

Understanding CRISPR-Cas9’s capacity to produce native overexpression (OX) alleles would accelerate agronomic gains achievable by gene editing. To generate OX alleles with increased RNA and protein abundance, we leveraged multiplexed CRISPR-Cas9 mutagenesis of noncoding sequences upstream of the rice *PSBS1* gene. We isolated 120 gene-edited alleles with varying non-photochemical quenching (NPQ) capacity in vivo—from knockout to overexpression—using a high-throughput screening pipeline. Overexpression increased OsPsbS1 protein abundance two- to threefold, matching fold changes obtained by transgenesis. Increased PsbS protein abundance enhanced NPQ capacity and water-use efficiency. Across our resolved genetic variation, we identify the role of 5′UTR indels and inversions in driving knockout/knockdown and overexpression phenotypes, respectively. Complex structural variants, such as the 252-kb duplication/inversion generated here, evidence the potential of CRISPR-Cas9 to facilitate significant genomic changes with negligible off-target transcriptomic perturbations. Our results may inform future gene-editing strategies for hypermorphic alleles and have advanced the pursuit of gene-edited, non-transgenic rice plants with accelerated relaxation of photoprotection.

## Teaser

Complex structural variants generated by CRISPR-Cas9 editing significantly increase native rice *PSBS* expression and activity.

## INTRODUCTION

Optimizing photosynthetic efficiency is one of the most promising routes to engineering more sustainable and productive crop varieties (*1*, *2*). Several recent breakthroughs have showcased the use of informed photosynthetic design principles to improve agronomic traits in the field. A photorespiratory bypass, made possible in part by cross-phyla overexpression of green algal glycolate dehydrogenase, increased field-grown biomass of *Nicotiana tabacum* by 20% (*3*) and enhanced relative fitness over the wild-type (WT) when grown under elevated temperatures (*4*). Overexpression of putative transcriptional regulators OsDREB1C (*5*) and zmm28 (*6*) have also shown substantial agronomic yield gains in model rice and elite maize varieties, respectively, although the causal effects on photosynthesis are still unclear. Transgenic overexpression of three highly conserved genes from *Arabidopsis thaliana* (*AtVDE*, *AtPsbS*, and *AtZEP*, hereafter VPZ), which are involved in photoprotection through non-photochemical quenching (NPQ), increased relaxation of but did not compromise NPQ under fluctuating light conditions, resulting in increases in tobacco biomass by 15% (*7*) and soybean seed yield by up to 20% (*8*) in small-scale field trials. The prospects for rationally designing photosynthesis in agronomic environments are bright.

However, these approaches relied on expression of foreign DNA, or transgenes, which incur regulatory complications and can be susceptible to gene silencing across generations (*9*). NPQ genes are found in all plants, and NPQ proteins are highly conserved in their function. We reasoned that altering endogenous gene expression could achieve similar agronomic gains without the need for persistent transgenes. As a proof of concept, we targeted the rice photosystem II (PSII) subunit S (*OsPSBS1*) gene, a core factor in high-light and fluctuating-light tolerance, to generate mutants with increased *OsPSBS1* expression and improved NPQ capacity.

Quantitative variation in rice *OsPSBS1* has already been observed between *japonica* (higher NPQ) and *indica* (lower NPQ) subspecies (*10*), potentially highlighting NPQ as a trait that has recently undergone selection. Transgenic overexpression of *PSBS* in rice has been shown to increase radiation-use efficiency in greenhouse-grown plants (*11*), and overexpression in tobacco has been implicated in increasing intrinsic water-use efficiency (iWUE) under red light (*12*). Rice is a diploid model C_3_ monocot with single-copy orthologs encoding VDE, ZEP, and a single, functional PsbS (OsPsbS1) (*13*). In addition, as a food crop that accounts for over 20% of all global calories consumed, rice is an impactful and genetically tractable target for improvement.

CRISPR-Cas9 has markedly expanded our capacity to produce targeted loss-of-function mutations. Recently, CRISPR-Cas9 editing of cis-regulatory elements has been used to decrease, rather than abolish, gene expression in tomato (*14*) and maize (*15*), improving fruit- and grain-yield–related traits. Similar multiplexed strategies have shown the potential of knocking out known cis-regulatory elements in pathogen susceptibility (*16*, *17*) or in identifying and editing cis-regulatory elements that constrain trade-offs between rice grain yield and plant architecture (*18*). Although newer toolkits such as the CRISPR-Cas12a promoter editing system (*19*) and approaches such as promoter swapping (*20*) show promise in advancing quantitative trait engineering efforts, finer understanding of ways to significantly increase gene expression without the use of persistent transgenes is still lacking.

To address these challenges, we established a high-throughput pipeline for screening novel alleles of *OsPSBS1* generated by CRISPR-Cas9 mutagenesis of upstream noncoding sequences (NCS). We generated a mutant library covering a large phenotypic range of *OsPSBS1* gene expression, including overexpression, with significant effects on NPQ and iWUE. Last, we identify themes in cis-regulation across our library that may inform gene-editing strategies for knockdown or overexpression of genes that affect other desirable plant traits.

## RESULTS

### High-throughput screening of edited, semidominant *OsPSBS1* promoter alleles

**Fig. 1.**
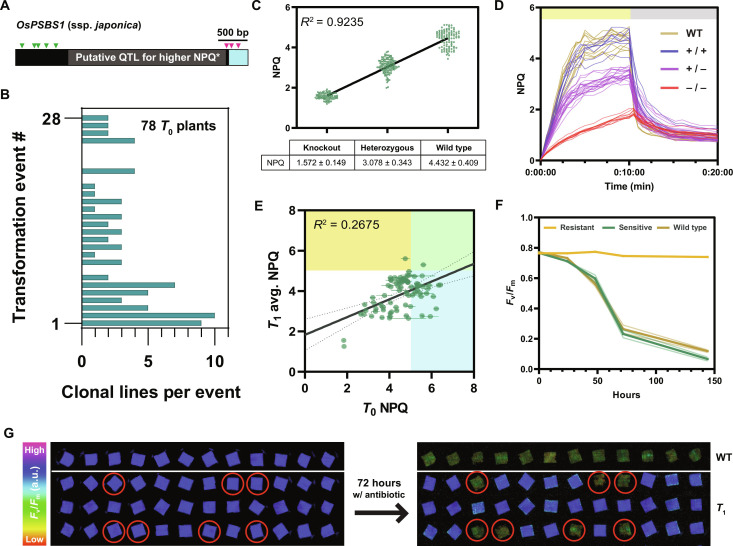
A high-throughput chlorophyll fluorescence to resolve *T*_1_ quantitative variation and *Cas9* transgene segregation. (**A**) Target gRNA sites (triangles) in distal (green) and proximal (magenta) regions upstream of *OsPSBS1*, with an insertion specific to ssp. *japonica* varieties marked in gray (*10*, *13*). (**B**) Seventy-eight clonal lines were regenerated across 23 independent events. QTL, quantitative trait locus; bp, base pairs. (**C**) Linear regression of maximum NPQ capacity after 10 min of blue light exposure at 1500 μmol photons m^−2^ s^−1^ in PsbS KO, heterozygous and WT lines (*n* = 95, 117, and 101 biological replicates respectively, across 26 segregating lines). (**D**) Chlorophyll fluorescence phenotyping of NPQ resolves homozygous alleles (+/+, WT-like, blue; −/−, KO, red) in 36 progeny of a segregating *T*_0_ parent (event #26) relative to WT (*n* = 12, brown). All replicates are shown. (**E**) Correlation of *T*_0_ max NPQ with the average max NPQ of its corresponding *T*_1_ alleles (*n* = 78 *T*_0_ plants, two to four biological replicates per *T*_1_ population). Individuals with NPQ exceeding +2 SD of WT in the *T*_0_ generation, *T*_1_ generation, or both generations are shown in cyan, yellow, and green respectively. (**F** and **G**) Addition of the plant selection antibiotic hygromycin to leaf punches phenotyped in (C) identifies sensitive individuals with inhibited *F*_v_/*F*_m_ that lack the *Cas9* transgene (circled). a.u., arbitrary units.

To generate novel cis-regulatory variation upstream of *OsPSBS1*, a CRISPR-Cas9 construct targeting eight specific and conserved rice guide RNA (gRNA) sites (table S1) was introduced into Nipponbare (ssp. *japonica*) rice calli via *Agrobacterium*-mediated transformation. The designed gRNAs target regions distal (green triangles) and proximal (magenta triangles) to the *OsPSBS1 gene*, avoiding a previously reported quantitative trait locus for increased NPQ activity in *japonica* rice varieties (Fig. 1A) (*10*, *13*). Twenty-three fertile, independent transformants were generated, and multiple plant lines were recovered from each transformed callus, when possible (Fig. 1B).

High-throughput in vivo chlorophyll fluorescence screening was used to resolve stable, heritable phenotypes across the 78 diploid *T*_0_ transformants, yielding up to 156 gene-edited alleles. Our approach leveraged the fact that *PSBS* is semidominant and shows a strong linear correlation between gene copy number and NPQ capacity (coefficient of determination, *R*^2^ = 0.9235) (Fig. 1C). Thus, in a segregating population, it is possible to resolve individuals with divergent, homozygous alleles by phenotype alone (Fig. 1D). This was important as phenotypes in the *T*_0_ generation may be somatic, and competition between alleles may mask changes in gene expression and activity in heterozygous individuals. Notably, maximum NPQ in the *T*_0_ generation was a poor predictor of stable *T*_1_ phenotypes (*R*^2^ = 0.2675). *T*_0_ phenotypes overestimated the number of putative *T*_0_ overexpressors (blue) and failed to identify a candidate stable *T*_1_ overexpressor line (yellow) (Fig. 1E).

This screen for NPQ phenotypes was extended to incorporate segregation of the hemizygous *Cas9* transgene. Leaf punches used to assess NPQ capacity (Fig. 1D) were treated with hygromycin, the antibiotic used for selection of transformants, and monitored for a decline in the maximum quantum efficiency of PSII (*F*_v_/*F*_m_), a common indicator of plant stress (Fig. 1, F and G). Obvious differences in resistance could be observed within 72 hours of incubation, with a decrease in *F*_v_/*F*_m_ of over 75% upon loss of *Cas9*, as verified by polymerase chain reaction (PCR) (fig. S1). By screening pools of 36 to 72 *T*_1_ progeny per *T*_0_ parent, it was possible to isolate multiple individuals harboring homozygous gene-edited alleles and lacking *Cas9* by Mendelian segregation of each trait, allowing us to rapidly identify fixed germplasm for further analysis in the *T*_2_ generation.

### Gene-edited, overexpression alleles are present, albeit rare

**Fig. 2.**
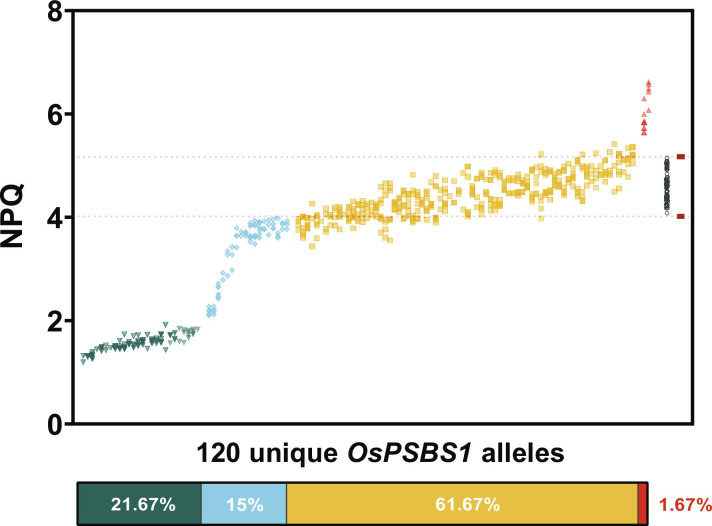
Maximum NPQ of 120 homozygous, gene-edited alleles spanning 78 *T*_0_ events. Maximum NPQ (*n* = 1 to 4 technical replicates each, two to eight biological replicates each) after 10 min of blue light exposure at 1500 μmol photons m^−2^ s^−1^. Each independent allele is sorted from lowest to highest average NPQ capacity. WT NPQ of 64 biological replicates spanning all phenotyping experiments is shown (gray, open circles on the right) with boundaries demarcating ±2 SD (red, dashed lines). Lines with all biological replicates below/above the WT boundary were binned by phenotype: knockout (KO; teal, inverse triangle), knockdown (KD; blue, diamond), WT-like (yellow, square), and overexpressor (red, triangle). Proportions of each observed phenotype are shown.

Putative homozygous alleles were identified via pairwise comparison between WT plants and progeny from a single *T*_0_ parent, as depicted in Fig. 1C. Of the 156 possible gene-edited alleles, 120 alleles were resolved by segregation of phenotypes across *T*_1_ progeny. To assess the variation in phenotypes across all alleles, maximum NPQ for all 120 homozygous alleles was plotted in Fig. 2.

Almost two-thirds of the 120 stable alleles isolated were WT-like in NPQ capacity (61.67%), with the second and third largest groups being knockout (KO; 21.67%) and KD (15%) phenotypes, respectively. Two independent overexpression (OX) alleles were isolated, comprising 1.67% of the total phenotypic variation.

### Representative alleles confirm phenotype-by-expression relationships

A panel of representative alleles spanning the KO to OX spectrum was selected to correlate observed NPQ phenotypes with gene expression and protein abundance. The full range of phenotypic diversity was present among clonal lines derived from a single transformation event (event 2), with the only other OX allele arising from event 19. Notably, event 2 also produced the greatest number of regenerated lines (Fig. 1A). This observation demonstrates that most gene editing occurred after initial selection of transformation events, and sister lines can experience divergent Cas9-editing trajectories.

**Fig. 3.**
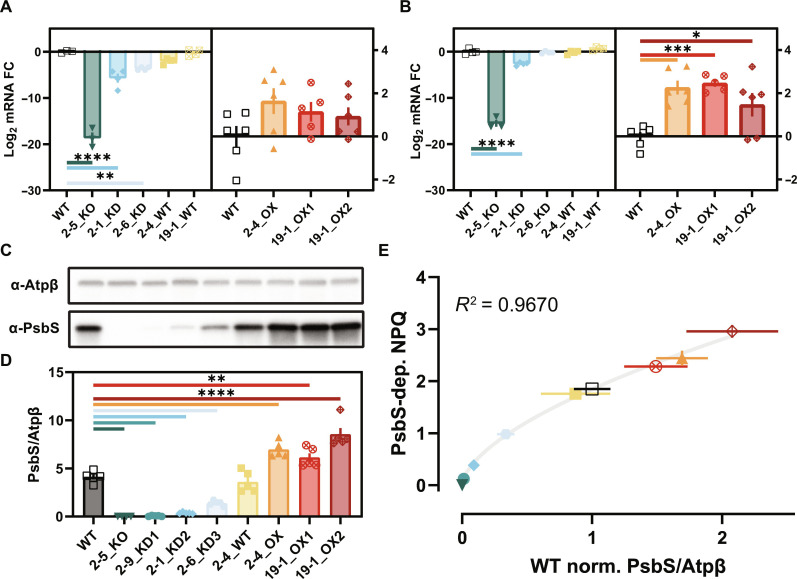
*OsPSBS1* transcript expression and protein abundance across eight representative alleles. Log_2_ relative *OsPSBS1* transcript quantification against WT, normalized to *OsUBQ* and *OsUBQ5*. Samples were collected across (**A**) morning and (**B**) evening experiments (*n* = 3 to 6 biological replicates, data ± SEM). Gene-edited alleles are ordered from lowest to highest total NPQ. FC, fold change. (**C**) Representative immunoblots (6 μg of total protein) of OsPsbS1 and the chloroplast loading control Atpβ. (**D**) Normalized OsPsbS1 immunoblot band intensity shown ± SEM across four (2-5_KO) or five (remaining genotypes) individual replicates each. (**E**) PsbS-dependent NPQ capacity (steady-state NPQ at 2000 μmol photons m^−2^ s^−1^ subtracted from the average residual NPQ in the 2-5_KO line). NPQ is plotted against WT-normalized OsPsbS1/Atpβ band intensity (means ± SEM shown). A logarithmic curve fit is shown in gray. For all panels, genotypes and replicates are shown: WT (black, open square), 2-5_KO (dark teal, inverse triangle), 2-9_KD1 (teal, circle), 2-1_KD2 (blue, diamond), 2-6_KD3 (light blue, hexagon), 2-4_WT (yellow, square), 2-4_OX (orange, triangle), 19-1_OX1 (red, crossed circle), and 19-1_OX2 (maroon, crossed diamond). Pairwise significance was determined by ordinary one-way analysis of variance (ANOVA) (𐓟 = 0.05) using Dunnett’s test for multiple comparisons against Nipponbare WT (*P ≤ 0.05, **P ≤ 0.01, ***P ≤ 0.001, and ****P < 0.0001).

Greenhouse-grown plants were sampled 1 hour after sunrise and 1 hour before sunset and analyzed for differences in *OsPSBS1* transcript levels. The event 2-5 KO line (hereafter referenced using the nomenclature “Event #-Line #_Phenotype”) was a useful internal control, because, unlike most other phenotypic KO alleles generated, 2-5_KO contains a large deletion that spanned the entire *OsPSBS1* coding sequence. Correspondingly, this allele had gene expression well below the threshold of detection by our *OsPSBS1*-specific primers [log_2_ fold change (log_2_FC) < −10]. We observed some stochasticity in gene expression across experiments; for example, there was a significant decrease in gene expression in 2-6_KD in the morning (Fig. 3A) but not the evening (Fig. 3B). Conversely, we found a statistically significant four- to eightfold increase in *OsPSBS1* transcript levels for OX lines in the evening dataset (Fig. 3B) but not the morning dataset, although trends in KD or OX lines across datasets were largely consistent.

OsPsbS1 protein abundance was determined by immunoblot, and a set of representative experiments is shown in Fig. 3C. Quantification of normalized band intensity across replicates confirmed protein abundances spanning 1.5 to 250% of WT OsPsbS1 levels (Fig. 3D), with all blots reported in fig. S2. The observed logarithmic best fit between protein abundance and PsbS-dependent NPQ (Fig. 3E) mirrors what has been reported in Arabidopsis (*21*).

### KO and KD alleles arise from variation in the 5′UTR

To assess the causal mutations underlying these phenotypes, the ~4.3-kb upstream cis-regulatory region was PCR-amplified and sequenced. One hundred seven of the 120 unique alleles (89.2%; Fig. 2) could be resolved by Sanger sequencing, as reported in fig. S3. Several alleles containing large deletions of the five distal gRNA target sites (Fig. 1A) were detected. These lines had NPQ capacity that was indistinguishable from WT (fig. S4). Thus, most of the observed phenotypic variation can be explained by variants within and near the 5′ untranslated region (5′UTR).

**Fig. 4.**
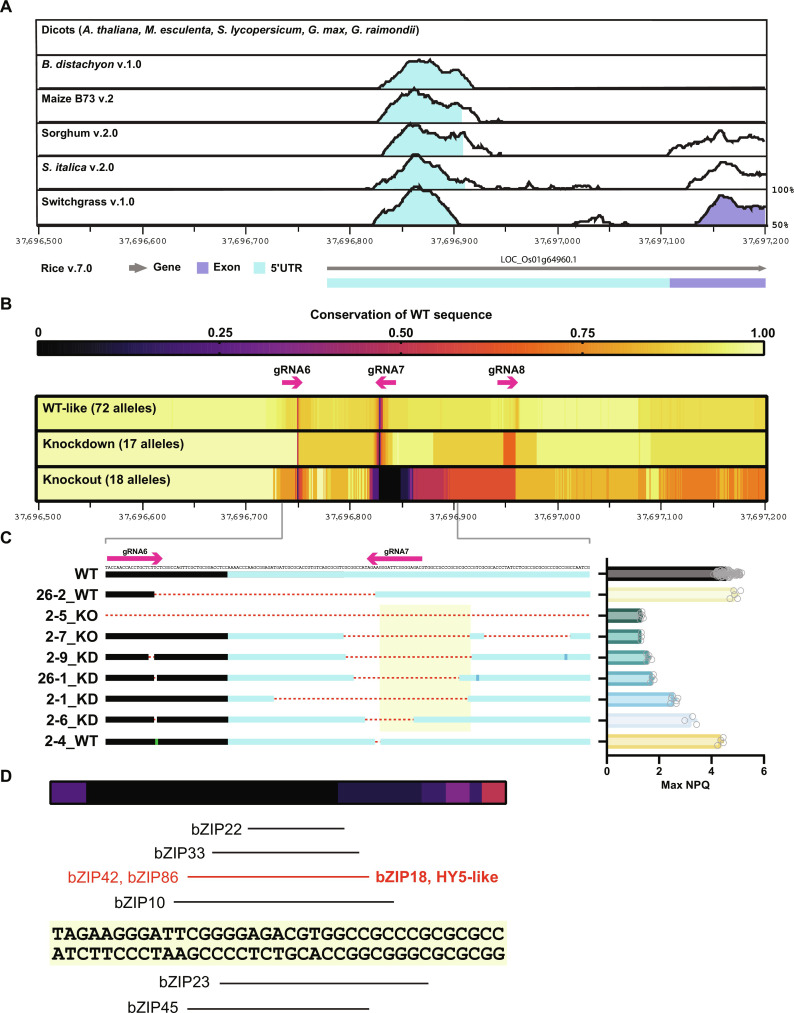
Aggregate analysis of gene-edited KO and KD alleles. (**A**) mVista alignments illustrating the percent sequence identity of the proximal *OsPsbS1* genomic locus (Chr01: 37,696,500 to 37,697,200) against five dicot and five monocot species, between 50 and 100% identity bounds. Shading indicates conservation exceeding 70% identity within the 5′UTR (cyan) or coding sequence (exon, purple) of the *OsPSBS1* gene. (**B**) Percent identity relative to the WT *OsPSBS1* sequence shown in (A) across gene-edited variants, as determined by multiple sequence alignment, and aggregated by NPQ phenotype. Target sites and directionality of gRNA are shown as magenta arrows. (**C**) A zoomed-in subset of representative KO and KD lines with small deletions spanning sites with low sequence conservation. Max NPQ phenotypes from (Fig. 2) are shown to the right of each allele (*n* = 3 to 5 biological replicates each, excluding WT). The yellow highlighted region in (C) is shown at higher nucleotide resolution in (**D**) relative to aggregate WT sequence conservation across KO alleles. Putative transcription factor binding sites predicted by PlantRegMap (*23*) are shown.

We used mVista (*22*) to assess variation in conserved noncoding sequences (CNSs) proximal to *OsPSBS1* across 10 representative species. We found no significant conservation across five dicot genome comparisons but saw consistent, high-confidence (>50%) conservation within the 5′UTRs of the *PSBS* gene in five representative monocot genomes (Fig. 4A).

Using our library of sequenced *OsPSBS1* NCS alleles (fig. S3), we assembled a phenotype-aggregated heatmap to correlate indels at the three proximal gRNA (gRNA6 to gRNA8) sites with phenotype (Fig. 4B). As expected, mutations are found most frequently at the 3′ NGG-proximal end of each gRNA, corresponding to the Cas9 protospacer adjacent motif (PAM) sequence. WT-like alleles showed that small indels can be tolerated without significant phenotypic cost. In contrast, shared large deletions within the monocot-conserved CNS were associated with KO phenotypes. KD alleles largely maintained the WT sequence within the CNS, but they had larger, variable indels (50 to 70% conservation) nearby that reduced but did not abolish *OsPSBS1* gene expression.

Several representative alleles with relatively small deletions were disaggregated to better understand the causal locus for *OsPSBS1* expression (Fig. 4C). The transcription start site appears to be completely dispensable and is not required for WT NPQ capacity. In contrast, deletions proximal to gRNA7 are associated with a range of KO and KD phenotypes, dependent on deletion size and location. We further interrogated the highlighted region in Fig. 4C, which encompassed the region of poor WT sequence conservation across KO lines (0 to 11%). Notably, this locus contains several high-confidence (*P* < 0.0001) binding sites for bZIP transcription factors (*23*), including bZIP18, a HY5-like ortholog (Fig. 4D).

### Complex structural variants underlie overexpression

Unexpectedly, the two OX alleles could not be resolved by PCR genotyping. To determine whether complex, structural variants were underlying these phenotypes, a homozygous 2-4_OX *T*_2_ line and segregating 19-1_OX *T*_1_ line were sequenced by long-read HiFi circular consensus sequencing (CCS; Pacific Biosciences). Reads were assembled de novo, mapped onto the Nipponbare *Oryza sativa* v7.0 reference genome (*24*), and used to produce dot plots showing sequence and structural variants (fig. S5, A and B).

Zooming in on the *OsPSBS1* locus on chromosome 1 revealed structural variation that was not observable at the whole-genome scale. The 2-4_OX line showed robust signatures of a ~252-kb inversion via dot plot (fig. S6A), which was further substantiated at the sequence level by looped, head-to-head single long-read sequences visualized by Integrative Genomics Viewer (fig. S6B) (*25*). The increased read depth, confirmed by quantitative PCR of genomic DNA copy number (fig. S6C), verified that the 2-4_OX allele was a 252-kb duplication/inversion.

**Fig. 5.**
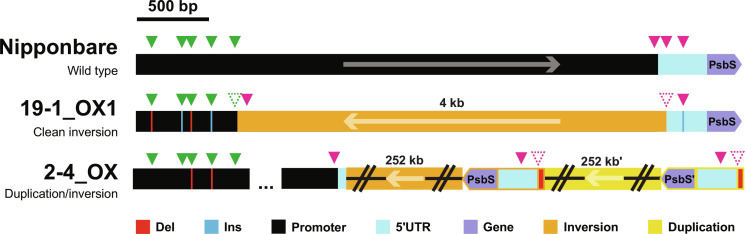
Long-read, whole-genome sequenced alleles driving *OsPSBS1* overexpression. Cartoon depictions of the stable 2-4_OX and 19-1_OX1 overexpression alleles relative to the WT promoter. Distal gRNA sites are marked by green triangles, proximal gRNA sites by magenta triangles, the 5′UTR in cyan, and the *OsPSBS1* gene in purple. One to 3-bp deletions (red lines) and insertions (blue lines) are shown relative to inversions (orange) and/or duplications (yellow). Structural variants that disrupt gRNA binding sites are shown with dashed triangles.

In contrast, the segregating 19-1_OX line showed no appreciable differences by dot plot (fig. S7A). However, local inspection revealed the presence of a smaller ~4-kb inversion with a Sniffles complex variant-called allele frequency of 0.361 (fig. S7B), which was successfully fixed to homozygosity (19-1_OX1). Despite this small genomic perturbation, we saw significant decreases in aboveground and grain biomass in a subset of dwarfed 19-1_OX progeny, hereafter referred to as 19-1_OX2 (fig. S8A). This was not true of the fixed 19-1_OX1 or 2-4_OX allele, which grew similarly to azygous WT controls (fig. S8B). Notably, isolating transgene-free 19-1 lines was confounded by the presence of 3 transferred DNA (T-DNA) insertions, as inferred from Mendelian segregation of antibiotic sensitivity (fig. S8C). In addition, while the 19-1_WT (*Cas9*^−^) and 19-1_OX1 (*Cas9*^+^) had 100% heritable alleles, 19-1_OX2 could not be fixed to phenotypic homozygosity even in the *T*_4_ generation (fig. S8D). It is unclear whether a linked, repetitive element found at low frequency by PacBio sequencing may be involved in the dwarf or NPQ OX phenotypes (fig. S7B), but the presence of dwarfed plants with WT NPQ capacity suggests that this phenotype is independent of the gain-of-function mutation. In summary, both stable *OsPSBS1* overexpression alleles (2-4_OX and 19-1_OX1) involve inversions upstream of the gene (Fig. 5).

### Transcriptomic analysis of the duplication/inversion

**Fig. 6.**
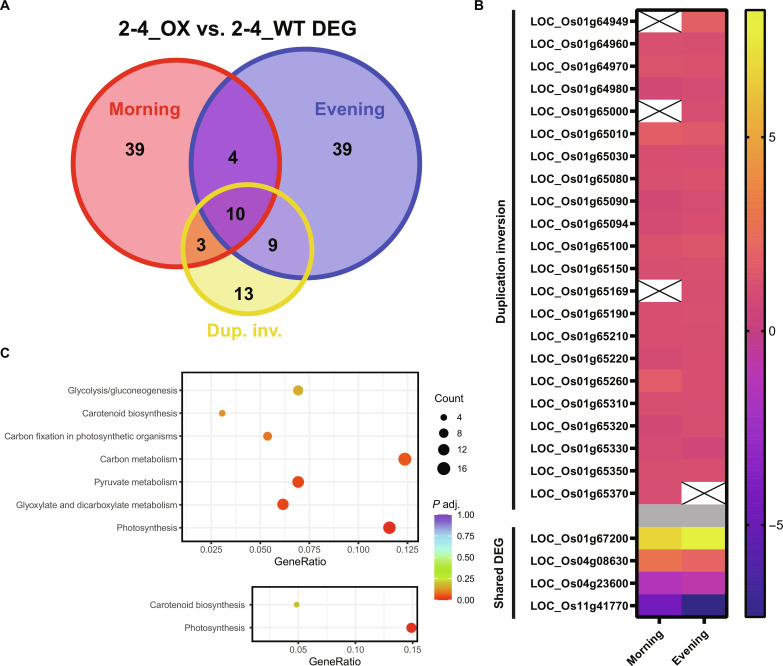
Transcriptome analysis of the 2-4_OX duplication/inversion. (**A**) Number of shared and unique differentially expressed genes (DEGs; adjusted *P* value of ≤0.1) between 2-4_OX and 2-4_WT alleles across morning (red) and evening (blue) samples relative to the 35 genes within the 252-kb duplication/inversion (yellow). (**B**) Log_2_FC gene expression of DEGs within (top) or outside (bottom) the duplication inversion across morning and evening datasets. X’s indicate genes that are not significantly differentially expressed (adjusted *P* value of >0.1) in their respective dataset. (**C**) Kyoto Encyclopedia of Genes and Genomes (KEGG) pathway enrichment (adjusted *P* value of ≤0.2) of all DEG (*P* value of <0.05) within the morning dataset (top) or across both morning and evening datasets (bottom).

The 252-kb duplication/inversion was a sizeable, marked genomic change generated by CRISPR-Cas9 mutagenesis. To determine the broader consequences of such a structural change, we performed an RNA sequencing (RNA-seq) experiment to assess the extent of differential gene expression between the 2-4_OX and 2-4_WT lines. In total, 104 differentially expressed genes (DEGs) were resolved using a generous adjusted *P* value (≤0.1), representing less than 0.2% of all genes within the genome. Twenty-two of all the significantly DEGs were contained within the duplication/inversion, representing 21.2% of all DEGs and 62.9% of all genes within the duplication/inversion (Fig. 6A).

Next, we assessed the log_2_FCs of individual DEGs. We observed a consistent twofold increase in expression (average log_2_FC =1.123 ± 0.255 SD) across the 22 differentially expressed duplication/inversion genes within either or both datasets. Of the remaining DEGs, only four were shared across both datasets, including the strongest up-regulated DEG: a ribosomal L13 family protein that was negligibly expressed in the 2-4_WT allele but actively expressed in the 2-4_OX allele (Fig. 6B). We leveraged Kyoto Encyclopedia of Genes and Genomes (KEGG) pathway enrichment to infer broader physiological consequences due to DEG up-regulation of *OsPSBS1* and nearby genes by the duplication/inversion. Of these pathways, photosynthesis and carbon assimilation–related metabolic pathways were significantly enriched (Fig. 6C).

### Varying PsbS abundance affects red light–dependent gas exchange phenotypes

Previous work has shown that transgenic overexpression of *PSBS* does not compromise steady-state CO_2_ assimilation in rice (*11*, *26*), but, in tobacco, it can increase maximum iWUE (*A*_n_/*g*_sw_) (*12*). To determine the extent to which these phenotypes are consistent in rice, seven phenotypically diverse genotypes were assessed for steady-state chlorophyll fluorescence and gas exchange phenotypes under increasing red light intensity. This required removing the contribution of stress-related effects on iWUE independent of plant genotype, constraining the minimum *F*_v_/*F*_m_ threshold of analyzed replicates to 0.803 (fig. S9A).

**Fig. 7.**
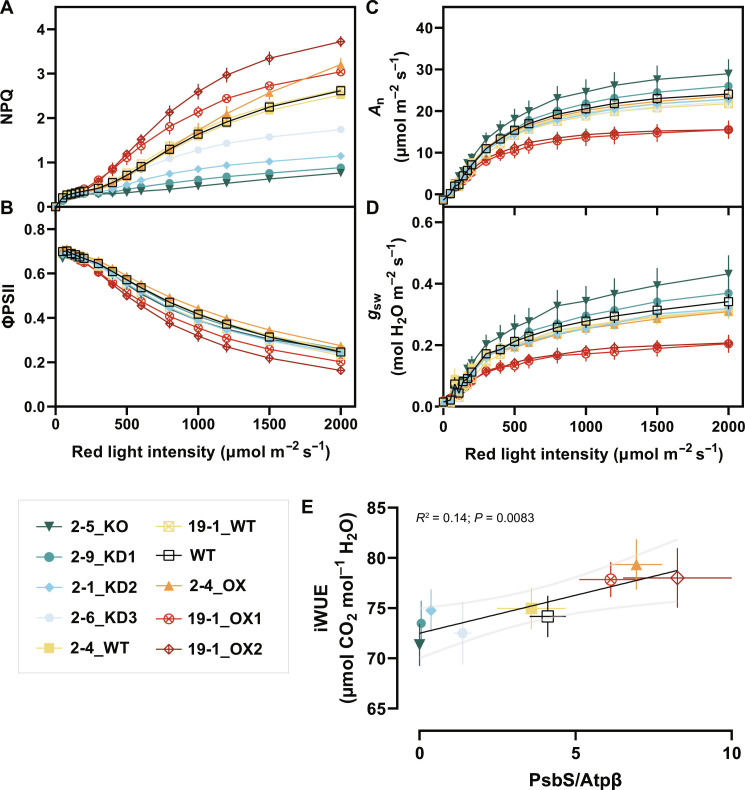
Concurrent chlorophyll fluorescence and gas exchange measurements of representative *OsPSBS1*-edited lines. (**A**) NPQ, (**B**) operating efficiency of PSII (ΦPSII), (**C**) CO_2_ assimilation (*A*_n_), and (**D**) stomatal conductance (*g*_sw_) as a function of incident red light on mature flag leaves (*n* = 4 to 7 biological replicates each, data ± SEM). (**E**) Linear regression of high-light iWUE (averaged *A*_n_/*g*_sw_, light intensity ≥ 500 μmol photons m^−2^ s^−1^) as a function of normalized OsPsbS1 protein abundance for each genotype (*n* = 4 to 7 biological replicates, means ± SEM shown). Genotypes are shown: WT (black, open square), 2-5_KO (dark teal, inverse triangle), 2-9_KD1 (teal, circle), 2-1_KD2 (blue, diamond), 2-6_KD3 (light blue, hexagon), 2-4_WT (yellow, square), 19-1_WT (yellow, crossed square), 2-4_OX (orange, triangle), 19-1_OX1 (red, crossed circle), and 19-1_OX2 (maroon, crossed diamond).

KD lines 2-9_KD1, 2-1_KD2, and 2-6_KD3 showed significantly lower NPQ (*P* < 0.0001), and overexpression lines 19-1_OX1 and 19-1_OX2 had significantly higher NPQ (*P* < 0.0001), at all light intensities greater than 500 μmol photons m^−2^ s^−1^ relative to the WT control. In contrast, 2-4_OX showed significantly higher NPQ only at 1500 and 2000 μmol photons m^−2^ s^−1^ (*P* < 0.0001) (Fig. 7A). Concurrent with the expanded higher NPQ capacity range, the 19-1_OX1 and 19-1_OX2 alleles exhibited a significantly lower operating efficiency of PSII (ΦPSII) (Fig. 7B), CO_2_ assimilation rate (*A*_n_; Fig. 7C), and stomatal conductance (*g*_sw_; Fig. 7D) compared to WT. The remaining event 2 genotypes showed little significant difference across ΦPSII, *A*_n_, or *g*_sw_, excluding a modest increase (*P* < 0.05) in CO_2_ assimilation rate in 2-5_KO at light intensities over 1200 μmol photons m^−2^ s^−1^ (Fig. 7C).

To assess differences in iWUE due to varying PsbS activity, all iWUE values under high light (≥ 500 μmol photons m^−2^ s^−1^) were averaged per replicate and plotted against WT-normalized PsbS protein abundance. A significant, positive correlation (*P* = 0.0083) between PsbS abundance and iWUE was observed, with a difference in average iWUE of ~11% between KO and OX lines (Fig. 7E).

### *Q*_A_ redox state is an inconsistent predictor of rice *g*_sw_

**Fig. 8.**
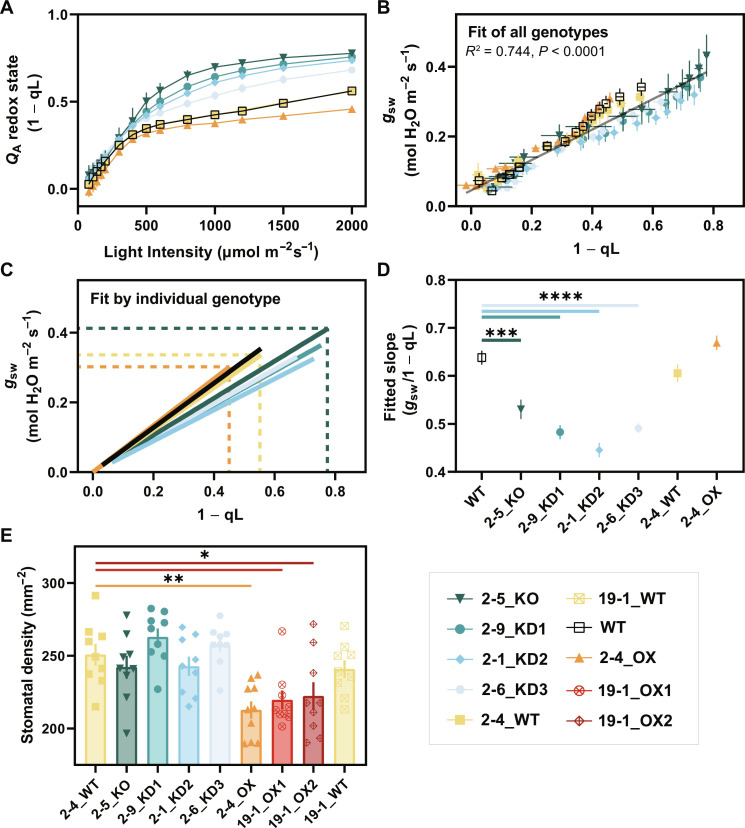
Correlation of *Q*_A_ redox state (1 − qL) and *g*_sw_ as a predictor of iWUE within event 2. (**A**) *Q*_A_ redox state (1 − qL) as a function of incident red light on mature flag leaves (*n* = 4 to 7 biological replicates, data shown ± SEM). (**B**) Linear regression of 1 − qL as a function of *g*_sw_ for all genotypes (*n* = 40 biological replicates) and (**C**) individuals genotypes (*n* = 4 to 7 biological replicates each) at all light steps exceeding 50 μmol m^−2^ s^−1^ (data shown ± SEM). Dashed lines indicate 1 − qL and *g*_sw_ values at 2000 μmol photons m^−2^ s^−1^ for the 2-5_KO, 2-4_WT, and 2-4_OX genotypes. (**D**) Slopes of linear regression lines by genotype (data shown ± SEM). (**E**) Stomatal densities of all gene-edited lines (*n* = 9 biological replicates each). Genotypes across (A to E) are shown: WT (black, open square), 2-5_KO (dark teal, inverse triangle), 2-9_KD1 (teal, circle), 2-1_KD2 (blue, diamond), 2-6_KD3 (light blue, hexagon), 2-4_WT (yellow, square), 19-1_WT (yellow, crossed square), 2-4_OX (orange, triangle), 19-1_OX1 (red, crossed circle), and 19-1_OX2 (maroon, crossed diamond). Pairwise significance in (D) and (E) was determined by ordinary one-way ANOVA (𐓟 = 0.05) using Dunnett’s test for multiple comparisons against Nipponbare WT or 2-4_WT and is denoted by asterisks (*P ≤ 0.05, **P ≤ 0.01, ***P ≤ 0.001, and ****P < 0.0001).

The increased iWUE phenotype observed in tobacco overexpressing *PSBS* was hypothesized to be mediated by the *Q*_A_ redox state, approximated by 1 − qL (*12*). We examined similar correlations between 1 − qL and *g*_sw_ using our event 2 panel, excluding possible off-target mutations affecting fitness and gas exchange phenotypes in the 19-1_OX lines (Fig. 7 and fig. S8). We observed robust, statistically significant differences in 1 − qL across all non-WT genotypes at light intensities above 500 μmol photons m^−2^ s^−1^ (Fig. 8A).

To interrogate the putative correlation, 1 − qL was plotted against stomatal conductance for all genotypes and light intensities. A significantly nonlinear (*P* < 0.0001) and relatively strong goodness of fit (*R*^2^ = 0.744) was observed (Fig. 8B), although the correlation was weaker than what was reported in tobacco (*R*^2^ = 0.98, *P* < 0.001) (*12*). We leveraged the extended dynamic range in 1 − qL phenotypes generated within this study to analyze linear regressions between 1 − qL and *g*_sw_ by individual genotype. While higher 1 − qL values were correlated with higher maximum *g*_sw_ values (dashed lines), the slopes of these regressions varied considerably across genotypes (Fig. 8C). There were statistically significant decreases in the relationship between *g*_sw_ and 1 − qL (i.e., slope) in all KO and KD lines relative to WT. In other words, larger changes in 1 − qL are associated with smaller relative changes in stomatal conductance in KO/KD but not WT/OX genotypes (Fig. 8D).

Last, we measured stomatal density across all gene-edited lines. Increases in PsbS protein abundance were correlated with statistically significant decreases in stomatal density of ~10% (0.01 < *P* < 0.05) (Fig. 8E), with no significant differences observed in KO or KD lines.

## DISCUSSION

As our understanding of basic biology grows, so does the potential for gene-editing solutions to address agricultural and medicinal challenges. The CRISPR toolkit has the potential to meet that need if the design principles modulating gene expression are well understood. Here, we show that multiplexed CRISPR-Cas9 mutagenesis of NCSs can achieve overexpression of endogenous genes and increase protein abundance to levels comparable to transgenic overexpression. Facilitated by a high-throughput screening pipeline for rapid detection of homozygous, *Cas9*-free progeny (Fig. 1), which revealed a diverse array of quantitative phenotypes (Fig. 2), these results have the potential to inform gene-editing strategies to optimize and fine-tune crop phenotypes.

The success of this screen was predicated on PsbS semi-dominance. However, this forward genetics approach came with inherent challenges. For example, the logarithmic relationship between PsbS abundance and NPQ (Fig. 3E) suggests that such a screen would enrich itself for KO/KD mutations, as modest gains in PsbS abundance will have negligible effects on overall NPQ capacity. While the two OX alleles identified in this study had significantly higher PsbS protein abundance and NPQ, it is possible that small-effect alleles are masked within the noise of WT-like biological variation (Fig. 2). Regardless, at a 1.67% observed frequency, it is important to recognize the constraints of random cis-regulatory element mutagenesis for OX. Advances in our understanding of cis-regulatory gene expression signatures may aid in generating and resolving small-effect alleles.

Consistent with a large body of literature, we find that overexpression of *PSBS* increases NPQ capacity (*11*, *12*, *27*). Overexpression of *OsPSBS1* in the 2-4_OX allele only increased steady-state NPQ capacity at very high light intensities (1500 and 2000 μmol photons m^−2^ s^−1^), similar to PsbS OX phenotypes in tobacco. Regardless, a strong correlation was observed between PsbS protein abundance and iWUE at light intensities as low as 500 μmol photons m^−2^ s^−1^ (Fig. 7E), suggesting that NPQ alone is not driving the iWUE response. Several converging lines of evidence, reviewed by Busch (*28*), suggest that the redox state of plastoquinone may be a well-correlated, red light–responsive regulator that might link photosynthesis and stomatal conductance through a yet unresolved signaling mechanism. Głowacka *et al.* (*12*) provided supporting correlations for this hypothesis through the overexpression of *AtPSBS* in tobacco, sidestepping pleiotropic effects observed in *g*_sw_ correlative studies that knocked down electron transport and CO_2_ assimilation (*29*, *30*). However, in rice, the increased iWUE phenotype is poorly explained by *Q*_A_ redox state, as assessed by 1 − qL (Fig. 8), which varied in its correlation with stomatal conductance across genotypes.

We also observed a marked decrease in stomatal density across all three OsPsbS1 OX lines (Fig. 8E), suggesting that the observed differences in iWUE cannot be explained by differences in stomatal aperture alone. There is abundant historical evidence that environmental cues such as light intensity, CO_2_ availability, and water stress regulate stomatal development (*31*, *32*). Is it possible that the overexpression of *PSBS* and its downstream effects on light harvesting intersect with some of these pathways? We cannot exclude the possibility that cumulative differences in excitation pressure at PSII during development (e.g., within vulnerable and developing leaves), potentially signaled by *Q*_A_ redox state, affect iWUE through a light-dependent effect on stomatal development and aperture. However, the lack of a correlative increase in stomatal density in any of the KO or KD lines suggests that 1 − qL alone is not a sufficient proxy for photosynthesis-dependent iWUE in our system (Fig. 8E). Morphological differences, such as the kinetically faster (*33*) dumbbell-shaped stomata in monocots (*34*), may also contribute to the reduced *Q*_A_-*g*_sw_ correlation and the relatively modest gains in iWUE (average, ~11% over KO and ~7% over WT) within the already more water-use efficient grasses.

While the observed phenotypes reinforce expected differences in PsbS-dependent traits, the most important results of this study relate to the structural variants underlying changes in gene expression. We found that distal regions of the *OsPSBS1* promoter, which are conserved between *indica* and *japonica* subspecies (table S1), were dispensable for expression of *OsPSBS1*, and much of the phenotypic variation observed was due to indels near and within the 5′UTR (Fig. 4B). The phenotypic consequences of editing monocot CNS underscore results from other leading works that have also converged on the importance and complexity of CNS editing (*19*, *35*).

Sequencing of our KO/KD alleles point to a possible role of a HY5-like ortholog, bZIP18, and other partially redundant bZIPs in being necessary for *OsPSBS1* expression (Fig. 4D). Rice bZIP18 may function similarly to Arabidopsis HY5 in partially regulating light-dependent gene expression (*36*), although whether deletion of this binding site also affects mature transcript stability or other factors that abolish gene expression remains to be determined. Regardless, the reduced genetic complexity (e.g., need for fewer guides and smaller editing region) presented by 5′UTR mutagenesis of sites flanking CNS may be an attractive approach for achieving controlled KD to bypass negative epistasis (*37*). The potential of 5′UTR mutagenesis for obtaining OX alleles, including the editing of competitive upstream open reading frames that may inhibit translation (*38*) or the use of prime-editing approaches (*39*) to introduce small enhancing GATC motifs downstream of the transcription start site (*40*), warrants further investigation.

Unexpectedly, the two OX alleles were caused by inversions upstream of *OsPSBS1* (Fig. 5). Allele 19-1_OX1 presents an interesting example as it carries a clean inversion between a pair of distal and proximal gRNAs that may readily be replicated or introgressed into other varieties. Unfortunately, the persistence of the high copy number T-DNA transgene in this line may contribute to the decreased overall fitness of 19-1_OX progeny (fig. S8). These pleiotropic off-target effects are also reflected in reductions in 19-1_OX gas exchange and ΦPSII phenotypes (Fig. 7, B to D) and the significantly increased NPQ capacity at lower light intensities (Fig. 7A), all of which have not been reported in transgenic rice PsbS overexpression experiments (*11*) or seen in the 2-4_OX line that displays a similar magnitude of PsbS1 overexpression (Fig. 3D). Future work to isolate this allele in a *Cas9*-free background or recapitulate the allele in other varieties of rice will be worthwhile to de-convolute PsbS abundance, photosynthesis, and gas exchange phenotypes.

The 2-4_OX 252-kb duplication/inversion was an unexpected but interesting result from this screen for transgene-free overexpression. However, such CRISPR-Cas9–induced variants are not atypical, as observed in multiple duplication/inversion events in mice (*41*), and a 338-kb tandem duplication in rice (*20*). Despite the significant genomic perturbation within the 2-4_OX allele, we observed negligible changes in global gene expression (Fig. 6) with most of the DEGs within the duplication/inversion itself. Correlating with copy number, we observe twofold overexpression of not just *OsPSBS1* but also the remaining genes within the duplication. The OX of the remaining 35 genes may result in other gain-of-function phenotypes that were not resolved within our NPQ-focused screen. KEGG enrichment of photosynthesis and carbon fixation metabolic pathways suggests that overexpression of *PSBS* and, potentially, its adjacent genes may have synergistic effects in contributing toward overall photosynthetic efficiency.

A revolution in long-read sequencing has revealed the pervasiveness of genomic structural variants. Complex structural variants including translocations, insertions, and inversions are persistent at both the population and pan-genome level as reported in tomato (*42*), rapeseed (*43*), maize (*44*), grapevine (*45*), and rice (*46*), affecting upward of 20% of all genes.

Much of the current understanding of structural variants is driven by the prevalence of these changes driving various human cancers and diseases (*47*–*49*). Unlike humans, however, plants exhibit a much greater tolerance to (and thus abundance of) structural variants that is likely driven in vivo by transposable elements and recombination, both documented as key drivers of crop domestication (*50*, *51*).

Chromosomal inversions have also been implicated in gene overexpression during the domestication of peach (*52*) and the complex rearrangements that underlie multiple myeloma (*53*). A 13-Mb inversion in highland maize has been implicated in adaptive, cold-responsive up-regulation of genes involved in photosynthesis (*54*). Eight large-scale inversions in domesticated cotton (*Gossypium hirsutum*) were strongly correlated with yield and fiber-related traits across F2 populations generated with the ancestral *Gossypium purpurascens* (*55*). In addition, a large 141-Mb inversion persists within elite barley lines across a 20 variety pan-genome, likely an unintended but conserved artifact of mutation breeding and domestication in the 1960s (*56*). While large-scale inversions may constrain recombination and drive speciation, they also significantly contribute to plant fitness and local adaptation (*57*).

Recently, Lu *et al.* (*20*) showed that CRISPR-Cas9 could be used to drive overexpression via promoter swapping, generating ~911-kb inversions in ~3% of transformed calli that increased gene expression of *OsPPO1* at varying frequencies. However, these inversions came at the cost of the opposite promoter, knocking out expression of the Calvin-Benson cycle protein 12 (*OsCP12*) gene (LOC_Os01g19740). In the case of our 19-1_OX1 allele, the other inversion breakpoint occurred only ~4 kb away, downstream of the 3′UTR of the neighboring gene (LOC_Os01g64930), and likely does not interfere with its expression. The work presented here, unbiased in its target design, reinforces and expands the genome-engineering potential of inversions for native gene overexpression.

We see significant promise in expanding beyond Targeting Induced Local Lesions in Genomes (TILLING) and local CRE-editing approaches to drive significant rather than small-effect changes in gene expression and phenotype. Efforts to increase the efficiency and frequency of complex structural variants such as by use of alternate Cas systems (*19*), paired prime-editing strategies (*58*), or DNA repair mutant backgrounds (*59*) may be enticing options to enrich for significant genomic disruptions and changes in endogenous gene overexpression. Guided design of inversions to minimize transcriptomic perturbations, such as inversions into transcriptionally active epigenetic marks (*60*), may also prove fruitful in increasing the frequency of rare overexpression alleles by inversions. In addition, investigating the potential of editing other NCS types (e.g., introns and 3′UTRs) may expand gene-editing opportunities to fine-tune expression for targets in gene-dense genomic loci. Further research identifying promising genomic markers for putative overexpression, such as synthetic enhancer signatures (*61*) and high expression 5′UTR motifs (*40*), is necessary to expand the reproducibility of these efforts. Applying these hypotheses to other NPQ genes, such as *OsVDE* and *OsZEP*, will bring us closer to optimizing photoprotection and native photosynthetic efficiency in crop plants.

## MATERIALS AND METHODS

### Plant material and growth conditions

Rice cultivar Nipponbare (*O. sativa* ssp. *japonica*) seeds were germinated on Whatman filter paper for 5 days at 100 μmol photons m^−2^ s^−1^ fluorescent light with a 14-hour day length (27°C day/25°C night temperature). Seedlings were transferred to soil composed of equal parts Turface and Sunshine Mix #4 (Sungro) and grown under seasonal day length (10 to 14 hours) in a south-facing greenhouse that fluctuated in temperature (38°C high/16°C low) and relative humidity (45 to 60%). Plants were fertilized with a 0.1% Sprint 330 iron supplement after transplanting at 2 weeks after germination and at the onset of grain filling at 10 weeks after germination and JR Peter’s Blue 20-20-20 fertilizer biweekly. Flats were kept full of water to mimic flooded growth conditions. Genotypes were randomized across flats and throughout the greenhouse to minimize positional effects. At the V4-5 leaf stage, *T*_1_ progeny and WT controls were assayed for differences in NPQ capacity and sensitivity to the selectable marker hygromycin.

To assess differences in photosynthetic efficiency and yield, gene-edited *T*_3_ plants were grown in a larger greenhouse with more homogenous light exposure and field-relevant growth conditions (40°C high/27°C low, 30 to 50% relative humidity) without supplemental light.

### gRNA design and cloning into Cas9 vector pRGEB32

Eight gRNA target sites were identified upstream of the functional *PSBS* ortholog in rice, *OsPSBS1* (LOC_Os01g64960), using CRISPR-P (crispr.hzau.edu.cn) (*62*) and a 1.5-kb region upstream of the *PSBS1* start codon in a draft genome of *O. sativa* ssp. *indica* cultivar IR64 (http://schatzlab.cshl.edu/data/rice/) (table S1) (*63*). The eight gRNA spacers were assembled into a DNA cassette interspersed with scaffolds and tRNA linkers for polycistronic gRNA expression as previously described (*64*) and synthesized (GenScript). The insert was cloned into the pRGEB32 rice *Agrobacterium*-mediated transformation vector (Addgene, plasmid no. 63142) via GoldenGate Assembly.

### Induction of embryogenic calli

Mature seeds of rice (*O. sativa* ssp. *japonica* cv. Nipponbare) were de-hulled and surface-sterilized for 20 min in 20% (v/v) commercial bleach (5.25% sodium hypochlorite) plus a drop of Tween 20. Three washes in sterile water were used to remove residual bleach from seeds. De-hulled seeds were placed on callus induction medium (CIM) [N6 salts and vitamins (*65*), maltose (30 g/liter), myo-inositol (0.1 g/liter), casein enzymatic hydrolysate (0.3 g/liter), l-proline (0.5 g/liter), l-glutamine (0.5 g/liter), 2,4-d (2.5 mg/liter), 6-Benzylaminopurine (BAP; 0.2 mg/liter), 5 mM CuSO_4_, and Phytagel (3.5 g/liter) (pH 5.8)] and incubated in the dark at 28°C to initiate callus induction. Six- to 8-week-old embryogenic calli were used as targets for transformation.

### *Agrobacterium*-mediated transformation

Embryogenic calli were dried for 30 min before incubation with an *Agrobacterium tumefaciens* EHA105 suspension (optical density at 600 nm = 0.1) carrying the cloned binary vector, pRGEB32_OsPsbS1_8xgRNA. After a 30-min incubation, the *Agrobacterium* suspension was removed. Calli were then placed on sterile filter paper, transferred to cocultivation medium [N6 salts and vitamins, maltose (30 g/liter), glucose (10 g/liter), myo-inositol (0.1 g/liter), casein enzymatic hydrolysate (0.3 g/liter), l-proline (0.5 g/liter), l-glutamine (0.5 g/liter), 2,4-d (2 mg/liter), thiamine (0.5 mg/liter), 100 mM acetosyringone, and Phytagel (3.5 g/liter) (pH 5.2)] and incubated in the dark at 21°C for 3 days. After cocultivation, calli were transferred to resting medium [N6 salts and vitamins, maltose (30 g/liter), myo-inositol (0.1 g/liter), casein enzymatic hydrolysate (0.3 g/liter), l-proline (0.5 g/liter), l-glutamine (0.5 g/liter), 2,4-d (2 mg/liter), thiamine (0.5 mg/liter), timentin (100 mg/liter), and Phytagel (3.5 g/liter) (pH 5.8)] and incubated in the dark at 28°C for 7 days. Calli were then transferred to selection medium [CIM and cefotaxime (250 mg/liter) and hygromycin B (50 mg/liter)] and allowed to proliferate in the dark at 28°C for 14 days. Well-proliferating tissues were transferred to CIM containing hygromycin B (75 mg/liter). The remaining tissues were subcultured at 3- to 4-week intervals on fresh selection medium. When a sufficient amount (about 1.5 cm in diameter) of the putatively transformed tissues was obtained, they were transferred to regeneration medium [MS salts and vitamins (*66*), sucrose (30 g/liter), sorbitol (30 g/liter), naphthaleneacetic acid (0.5 mg/liter), BAP (1 mg/liter), and cefotaxime (150 mg/liter)] containing hygromycin B (40 mg/liter) and incubated at 26°C, 16-hour light, 90 μmol photons m^−2^ s^−1^. When regenerated plantlets reached at least 1 cm in height, they were transferred to rooting medium [MS salts and vitamins, sucrose (20 g/liter), myo-inositol (1 g/liter), and cefotaxime (150 mg/liter)] containing hygromycin B (20 mg/liter) and incubated at 26°C under conditions of 16-hour light (150 μmol photons m^−2^ s^−1^) and 8-hour dark until roots were established and leaves touched the Phytatray lid. When possible, multiple regenerants per calli were recovered. Plantlets were then transferred to soil.

### Chlorophyll fluorescence measurements of NPQ

Leaf punches were sampled from mature, fully developed leaves at leaf stage V3-5 and floated on 270 μl of water in a 96-well plate. Plates were dark acclimated for at least 30 min before analysis. In vivo chlorophyll fluorescence measurements were determined at room temperature using an Imaging-PAM Maxi (Walz) pulse-amplitude modulation fluorometer. Fluorescence levels after dark acclimation (*F*_o_, *F*_m_) and during light acclimation (*F*_o_′, *F*_m_′) were monitored in two ways.

To resolve phenotypically segregating *OsPSBS1* gene-edited alleles from a single parent, a single leaf punch at the V3 stage was exposed to a 4-min period of high-intensity actinic light (1500 μmol photons m^−2^ s^−1^) using periodic saturated pulses. Putative homozygous lines were identified as those progenies in the top and bottom 10% of total NPQ. The leaf punches were then used to determine hygromycin sensitivity and *Cas9* transgene segregation as described.

Candidates above lacking *Cas9* when possible were resampled at the V5 leaf stage, phenotyping two leaf punches from two mature leaves per plant to compare NPQ relative to Nipponbare WT plants. NPQ was quantified during a 10-min period of high-intensity actinic light (1500 μmol photons m^−2^ s^−1^) and 10-min dark relaxation (0 μmol photons m^−2^ s^−1^) using periodic saturating pulses. NPQ in both cases was calculated (Eq. 1).

$$\text{NPQ}=\left[\left(F_{m}-F_{m}\prime \right)/F_{m}\prime \right]$$ (1)

### Hygromycin sensitivity assay for high-throughput *Cas9* transgene detection

Floated leaf punches assayed for NPQ capacity were used to determine hygromycin sensitivity and segregation of the transgene. Hygromycin B (50 mg/ml; 1× phosphate-buffered saline) was added to each well to antibiotic with a final concentration of 20 μg/ml. Plates were incubated under rice germination conditions for 3 days, following which the Imaging-PAM Maxi (Walz) was used to identify differences in the maximum efficiency of PSII (*F*_v_/*F*_m_) after 30 min dark acclimation using (Eq. 2).

$$F_{v}/F_{m}=\left(F_{m}-F_{o}\right)/\left(F_{m}\right)$$ (2)

Sensitive, transgene-free plants had a decline in *F*_v_/*F*_m_ of >0.3 to 0.4, whereas transgenic plants maintained a WT *F*_v_/*F*_m_ of ~0.7 to 0.8.

### Total RNA/protein extraction of representative lines with varying NPQ

Leaf tissue (2 cm) from the youngest fully developed leaf was collected and flash-frozen in ribonuclease-free, deoxyribonuclease (DNAse)–free tubes containing Lysing Matrix D (FastPrep-24) at midday. Leaf tissue was ground on dry ice using a FastPrep-24 5G High-Speed Homogenizer (6.0 m/s for 2 × 40 s, MP Biomedical). Protein and mRNA were extracted from the same leaf sample (NucleoSpin RNA/Protein kit, REF740933, Macherey-Nagel GmbH & Co., Düren, Germany).

### qRT-PCR of *OsPSBS1* relative to two reference genes

Extracted mRNA was treated with DNase (ThermoFisher Scientific) and transcribed to cDNA using Omniscript Reverse Transcriptase (QIAGEN) and a 1:1 mixture of random hexamers and oligo(dT) as recommended by the manufacturer. Quantitative reverse transcription (qRT)–PCR using previously published primers (*67*, *68*) was used to quantify *OsPSBS1* transcripts relative to *OsUBQ* and *OsUBQ5* transcripts with technical duplicates using published methods to normalize qRT-PCR expression to multiple reference genes (*69*, *70*). Samples were run on a 7500 Fast Real-Time PCR system (Applied Biosystems, Gent, Belgium) in a total volume of 20 μl using 4 μl of 1:10 diluted cDNA. Primers were empirically validated on a five-step dilution series of WT cDNA (1:1 to 1:81). All final primer pairs had an amplification efficiency between 90 and 105% and linear amplification within the dynamic range tested. A single peak in melt-curve analysis was observed for each gene of interest, verifying specificity of the amplicon. The primer sets were similarly efficient and specific for *OsPSBS1* copy number experiments of RNA-free genomic DNA. Primers and primer efficiencies are shown in table S2.

### Immunoblotting of whole-leaf protein extracts

Precipitated protein was resuspended in the supplied protein solubilization buffer with Tris(2-carboxyethyl)phosphine (PSB-TCEP) and quantified using a trichloroacetic acid colorimetric assay (*71*). Samples containing 6 μg of total protein were resolved using pre-cast SDS–polyacrylamide gel electrophoresis Any KD gels (Bio-Rad), transferred to a polyvinylidene difluoride membrane (Immobilon-FL, 0.45 μm, Millipore) via wet transfer, and blocked with 3% nonfat dry milk for immunodetection. Membranes were cut and incubated with the following antibodies. A rabbit polyclonal antibody raised against sorghum PsbS (𐓟-SbPsbS, DEEVTGLDKAVIQPGKGFRGALGLSE-Cys) was produced by Pacific Immunology, shared by S. J. Burgess (University of Illinois), and used at a 1:2500 dilution. A rabbit polyclonal antibody raised against a synthetic peptide of the β subunit of ATP synthase (Atpβ) was obtained from Agrisera (catalog no. AS05 085) and used at 1:10,000 dilution. After incubation with an horseradish peroxidase–conjugated, anti-rabbit secondary antibody from GE Healthcare (1:10,000 dilution), bands were detected by chemiluminescence using SuperSignal West Femto Maximum Sensitivity Substrate (Thermo Fisher Scientific). Protein bands were quantified by densitometry with ImageQuant TL software (version 7.0 GE Healthcare Life Sciences, Pittsburgh, PA, USA). PsbS abundance was quantified relative to WT via ImageLab.

### PCR genotyping of transgene-free, edited lines

Leaf tissue (50 mg) was ground via bead beating (Lysing Matrix D, FastPrep-24), and genomic DNA was extracted in 2× CTAB (cetyltrimethylammonium bromide) buffer at 65°C for 15 min. DNA was separated via chloroform phase separation and precipitated using isopropanol. The pellet was washed briefly in 70% ethanol before being dried and resuspended in 1× TE (Tris-EDTA) buffer.

Two overlapping regions spanning the eight gRNA target sites were PCR-amplified via Phusion High-Fidelity PCR using 5× GC-rich buffer (table S3). PCR products were amplified from at least two putative homozygotes per line and sequenced by Sanger Sequencing. Contigs of the ~4.3-kb upstream region were assembled using Snapgene and analyzed by multiple sequence alignment.

### In-parallel gas exchange and chlorophyll fluorescence analysis

Photosynthetic gas exchange dynamics were measured on the youngest, fully expanded flag leaf of 12-week-old flowering rice plants. Gas exchange measurements were performed using an open gas exchange system (LI6800, LI-COR, Lincoln, NE, USA) equipped with a 2-cm^2^ leaf chamber and integrated modulated fluorometer and normalized to leaf area. Whole plants were low light acclimated for 1 to 2 hours to mitigate afternoon depression of photosynthesis and dark acclimated for at least 30 min to allow for concurrent phenotyping of gas exchange (e.g., *A*_n_ and *g*_sw_) and chlorophyll fluorescence parameters (e.g., *F*_v_/*F*_m_ and NPQ). For all measurements, the chamber conditions were set to 400 parts-per-million CO_2_, 27°C chamber temperature, 1.3- to 1.5-kPa vapor pressure deficit of the leaf, flow rate of 500 μmol s^−1^, and a fan speed of 10,000 rpm. Samples were assayed within the boundaries of ambient daylength (8 a.m. to 5 p.m.).

Steady-state photosynthesis and stomatal conductance were monitored in response to changes in red light intensity (100% red light-emitting diodes; λ_peak_, 630 nm). Light intensity was varied from 0, 50, 80, 110, 140, 170, 200, 300, 400, 600, 800, 1000, 1200, 1500, and 2000 μmol photons m^−2^ s^−1^ with 10 to 20 min of acclimation per light step. Steady state was reached when the stomatal conductance, *g*_sw_, maintained a slope less than 0.005 ± 0.00025 SD over a 40-s period and when net assilimation rate, *A*_n_, showed variation less than 0.5 ± 0.25 SD over a 20-s period. Net assimilation rate, stomatal conductance, and intracellular (CO_2_) were logged. A saturating pulse was then applied to collect all relevant chlorophyll fluorescence parameters using a multiphase flash routine. In addition to NPQ and *F*_v_/*F*_m_, we assessed ΦPSII, iWUE, and *Q*_A_ redox state (1 − qL) (Eqs. 3 to 5). The derivation of 1 − qL assumes a “lake” model for photosynthetic antenna complexes.

$$\text{ΦPSII}=\left(F_{m}\prime -F_{s}\prime \right)/F_{m}\prime$$ (3)

$$\text{iWUE}=A_{n}/g_{\text{sw}}$$ (4)

$$1-\text{qL}=1-\left(F_{q}\prime /F_{v}\prime \right)/\left(F_{o}\prime /F_{s}\prime \right)$$ (5)

### PacBio long-read whole-genome sequencing and analysis

Leaf tissue used for high–molecular weight genomic DNA (HMW gDNA) extraction was dark starved for 4 days before being sampled and flash-frozen in liquid nitrogen. DNA was isolated using the NucleoBond HMW DNA kit (TakaraBio, catalog no. 740160.2) with the following modifications: Plant leaves were ground by pestle and mortar under liquid nitrogen, where 1 g of ground leaf tissue was resuspended in 2.5 times the amount of recommended lysis buffer and incubated in a 50°C water bath for 4 hours. The amount of Binding Buffer H2 was also proportionately increased. HMW gDNA was resuspended by gentle pipetting in water and assessed for quality by Femto Pulse Analysis (median fragment length, 13 to 15 kb).

HMW gDNA was pooled and sequenced using a PacBio Sequel II (QB3 Genomics, UC Berkeley, Berkeley, CA, RRID:SCR_022170) by HiFi CCS on a single 8M SMRTcell. Untrimmed reads for overexpression lines N2-4_OX and N19-1_OX were first quality checked using FastQC (www.bioinformatics.babraham.ac.uk/projects/fastqc/). Reads were mapped to the *O. sativa* v7.0 reference genome (*24*) using pbmm2, a minimap2 wrapper by Pacific Biosciences specifically for HiFi reads, and assembled using pbIPA, Pacific Biosciences phased assembler (https://github.com/PacificBiosciences/pbbioconda). Sniffles (https://github.com/fritzsedlazeck/Sniffles) and pbsv (https://github.com/PacificBiosciences/pbbioconda) were used to identify structural variants from mapped reads. D-Genies (*72*) was used to align the de novo assembly to the reference genome and generate dot plots. Integrative Genomics Viewer (*25*) was used to visualize mapped reads, aligned assemblies, and sniffles structural variation output.

### Whole transcriptome sequencing

RNA-seq was performed by Novogene (www.novogene.com). Briefly, mRNA was purified from total RNA using poly-T oligo-attached magnetic beads for 150–base pair paired-end sequencing on a NovaSeq6000. Raw data (raw reads) in fastq format was first processed using Novogene perl scripts. Reference genome and gene model annotation files were downloaded from Phytozome (*24*). Paired-end clean reads were aligned to an index of the reference genome built using Hisat2 v2.0.5 (*73*). The mapped reads of each sample were assembled by StringTie v1.3.3b (*74*) in a reference-based approach. FeatureCountsv1.5.0-p3 (*75*) was used to count the reads numbers mapped to each gene. Then, FPKM (fragments per kilobase of exon per million mapped reads) of each gene was calculated on the basis of the length of the gene and reads count mapped to this gene. Fold change differences were calculated using the DESeq2 package (*76*, *77*). The resulting *P* values were adjusted using the Benjamini and Hochberg’s approach for controlling the false discovery rate. Genes with an adjusted *P* value of ≤0.10 found by DESeq2 were assigned as differentially expressed. Last, we used the clusterProfiler R package to test the statistical enrichment of differential expression genes in KEGG pathways (*78*).

### Stomatal density measurements

Stomatal density was measured on the abaxial side of the fourth fully expanded true leaf with method adapted from Karavolias *et al.* (*79*). Briefly, epidermal impressions of nine biological replicates were taken from the widest section of the leaves. Images of each impression were taken using a Leica DM5000 B epifluorescent microscope at ×10 magnification. Three images were captured from each impression. The number of stomata in a single stomatal band was counted and divided by the area of the band to calculate stomatal density. The stomatal density calculated from the three images was averaged to represent the stomatal density of each biological replicate.

### Statistical analysis

All statistical analysis was performed using GraphPad Prism version 10.0.0 for Windows (www.graphpad.com). Pairwise significance was determined by ordinary one-way or two-way analysis of variance (ANOVA; 𐓟 = 0.05) using Dunnett’s test for multiple comparisons against the designated WT control. Linear and logarithmic regressions were fitted using individual replicates, with average values plotted for clarity.
