# Supplementary material for: Multiplexed CRISPR-Cas9 mutagenesis of rice PSBS1 noncoding sequences for transgene-free overexpression
Source: Sci Adv. 2024 Jun 7;10(23):eadm7452. doi: 10.1126/sciadv.adm7452 (PMC11160471; doi:10.1126/sciadv.adm7452)
Supplement: Supplementary file 1 — Figs. S1 to S9 Tables S1 to S3 [file sciadv.adm7452_sm.pdf]

Science Advances
AAAS

Supplementary Materials for

Multiplexed CRISPR-Cas9 mutagenesis of rice *PSBS1* noncoding sequences for transgene-free overexpression

Dhruv Patel-Tupper *et al.*

Corresponding author: Krishna K. Niyogi, niyogi@berkeley.edu

Sci. Adv. **10**, eadm7452 (2024)
DOI: 10.1126/sciadv.adm7452

This PDF file includes:

Figs. S1 to S9
Tables S1 to S3

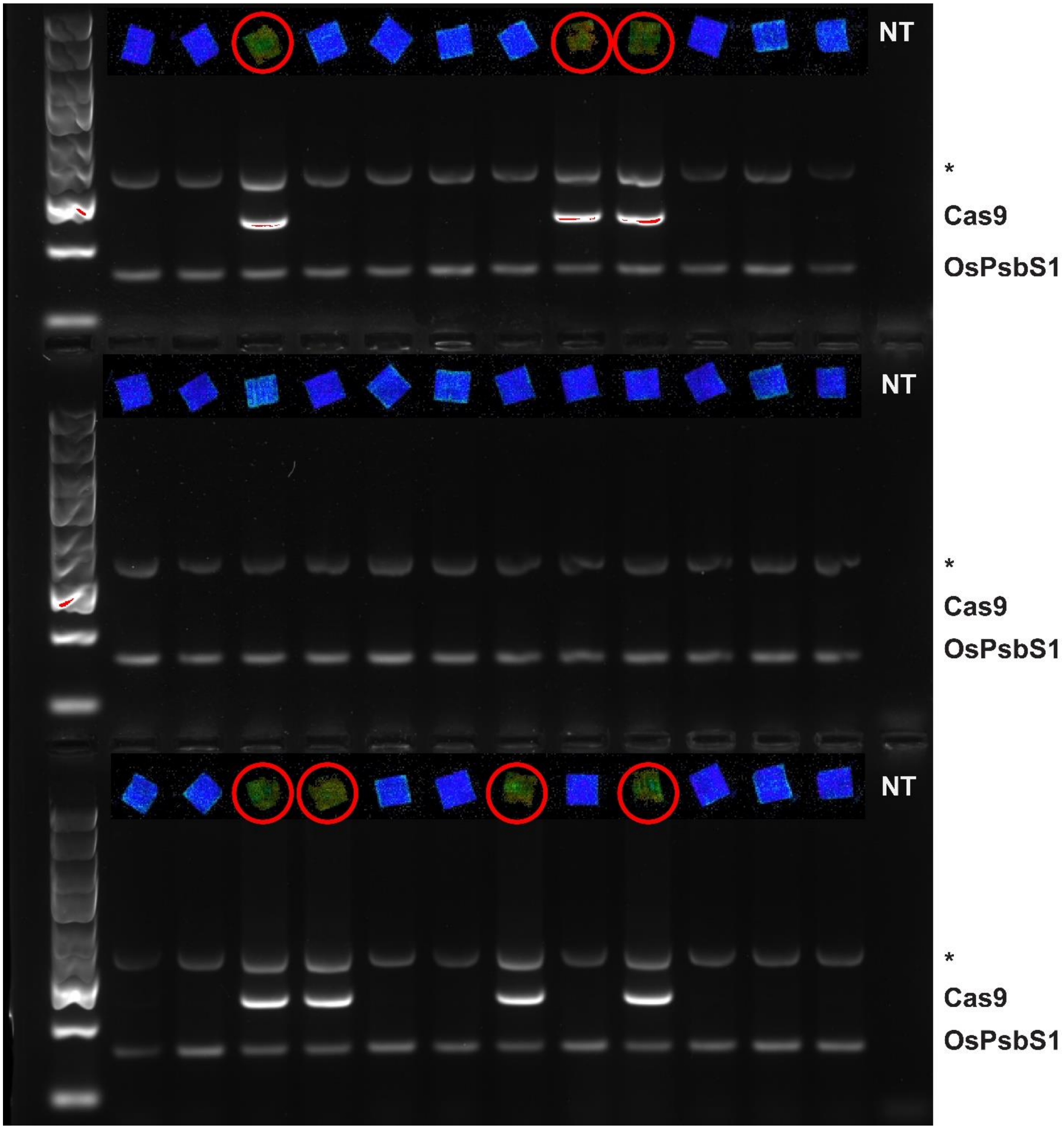

Fig. S1. Transgene genotyping of hygromycin-resistant and -sensitive plants.

Gel electrophoresis results of pooled PCRs amplifying the *Cas9* transgene and a region within the *OsPSBS1* coding sequence. Corresponding leaf punches from (Fig. 1F) are superimposed above each lane, with antibiotic-sensitive leaves with reduced F_v/F_m circled in red. A non-specific background band is noted by an asterisk (*).

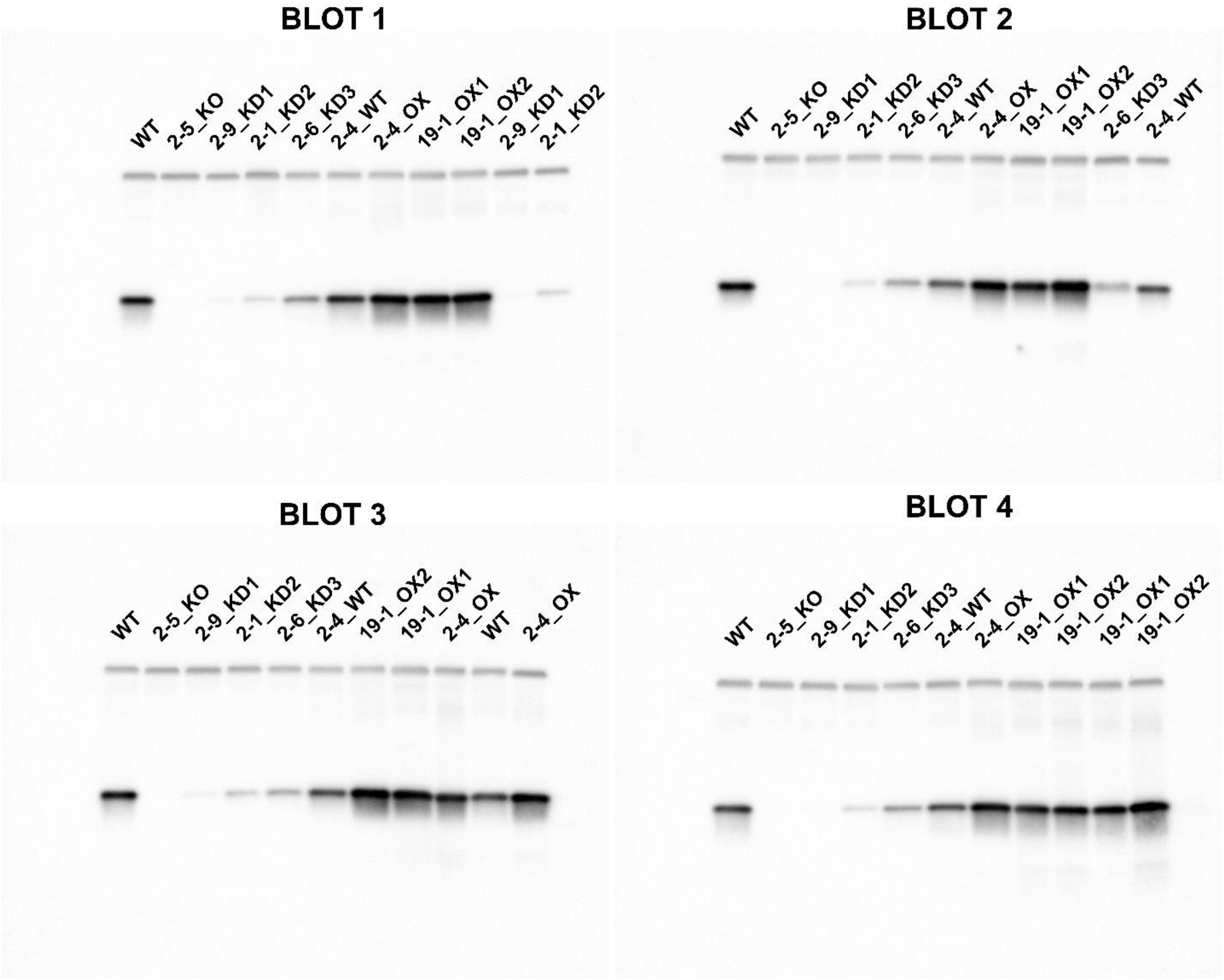

Fig. S2. All immunoblots used for quantification of Atpβ and PsbS.

Immunoblots detecting chloroplastic AtpB (1:10000, top band) and PsbS (1:2500, bottom band) on 4 independent blots, representing 4-5 biological replicates per genotype at a 5 s exposure time. 6 µg of total protein was loaded into each well.

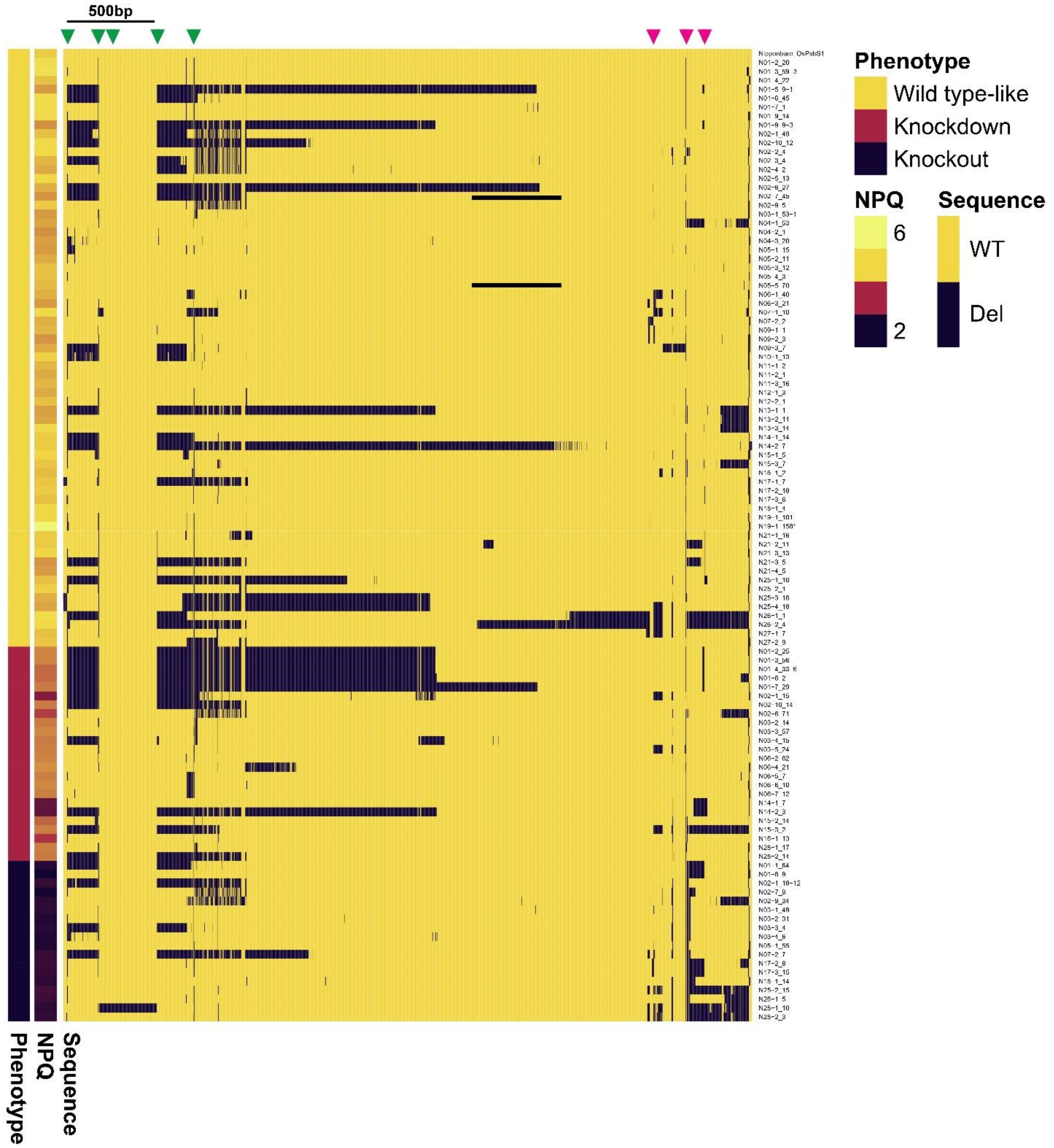

Fig. S3. Individual, sequenced *OsPSBS1* promoter alleles sorted by NPQ phenotype.

Sequence identity of the 107 PCR-amplified, gene-edited alleles with deletions (indigo) relative to the Nipponbare *OsPSBS1* gene (top). Target gRNA sites (triangles) in distal (green) and proximal (magenta) regions upstream of *OsPSBS1* are also shown. Alleles are sorted by event number and binned by relative phenotype, with the maximum NPQ capacity of each allele also reported. The asterisked allele (19-1_158*) corresponds to the 19-1_OX1 structural variant resolved by long-read, whole-genome sequencing.

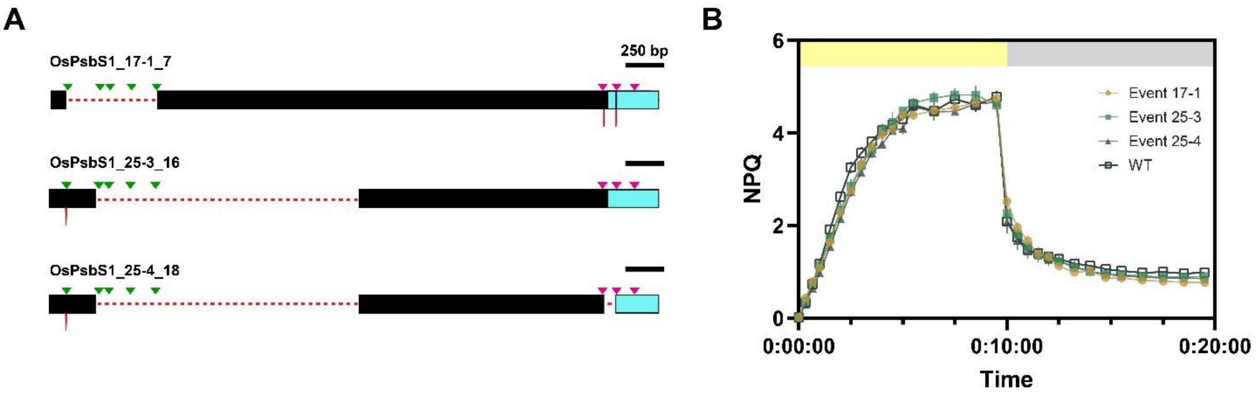

Fig. S4. Large distal deletion alleles and associated NPQ capacity.

(**A**) Three unique cis-regulatory mutant alleles with large deletions (red dashed lines) at distal gRNA sites (green triangles) mapped onto the Nipponbare promoter. The 5'UTR is shown in cyan. The scale bar is 250 bp. (**B**) NPQ kinetics of large distal deletion lines in (A) as follows: Event17-1_7 (brown, circle), Event 25-3_16 (teal, square), Event 25-4_18 (gray, triangle), Nipponbare WT (black, open square). Data shown ± SEM.

A

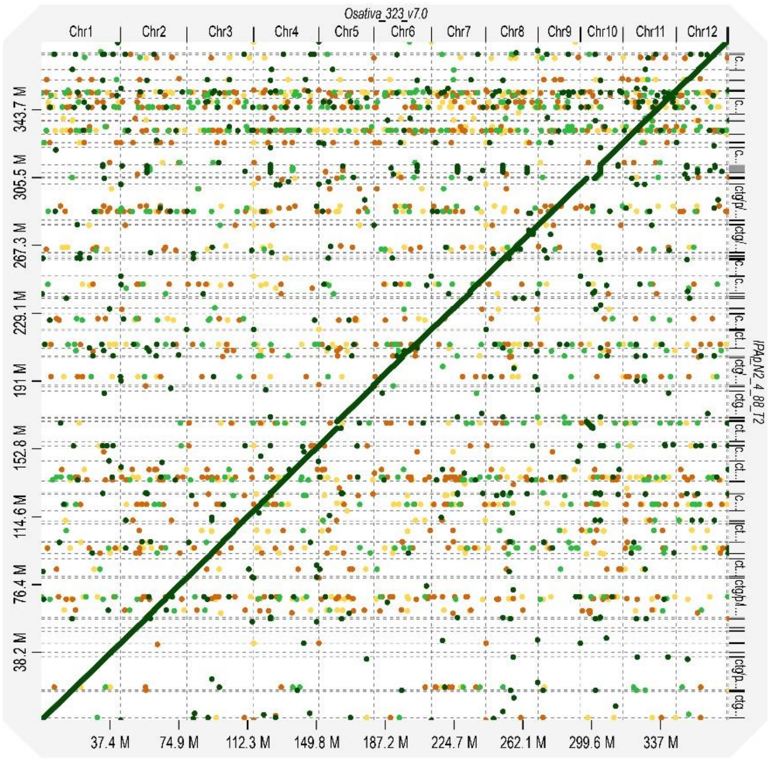

B

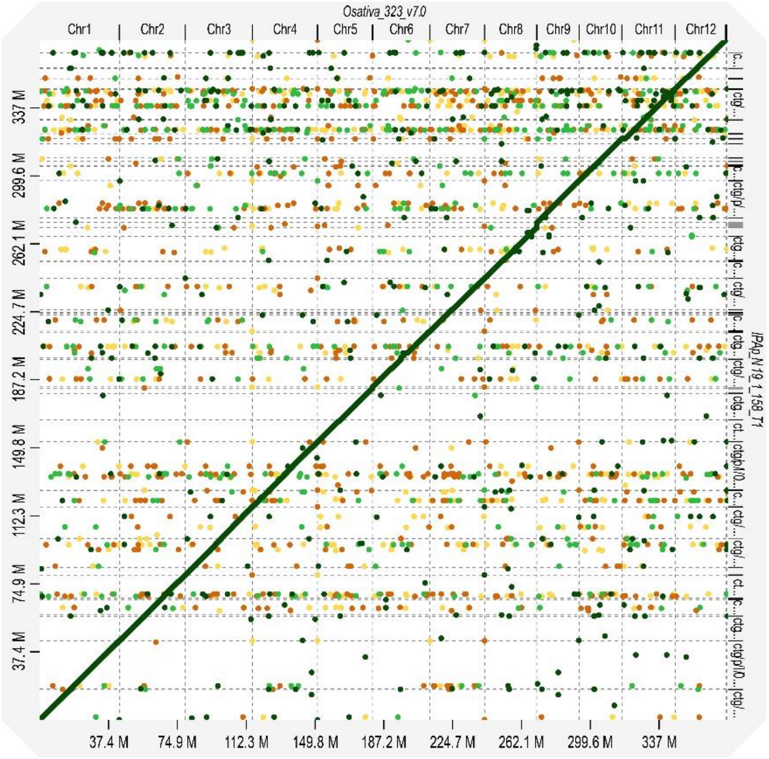

Fig. S5. Dot plots of Pacbio long-read sequencing of overexpression alleles.

(**A**) Dot plot of 2-4_OX and (**B**) 19-1_OX, plotting sequenced OX variants (y-axis) against the reference genome (x-axis). Colors indicate the strength of matched sequences, with yellow being the lowest confidence (0-25%) and dark green being the highest (75-100%). Continuity on the intersecting axis (diagonal green) indicates high similarity between both genomes, with chromosome-level insertions, deletions, and structural variants shown by gaps in continuity.

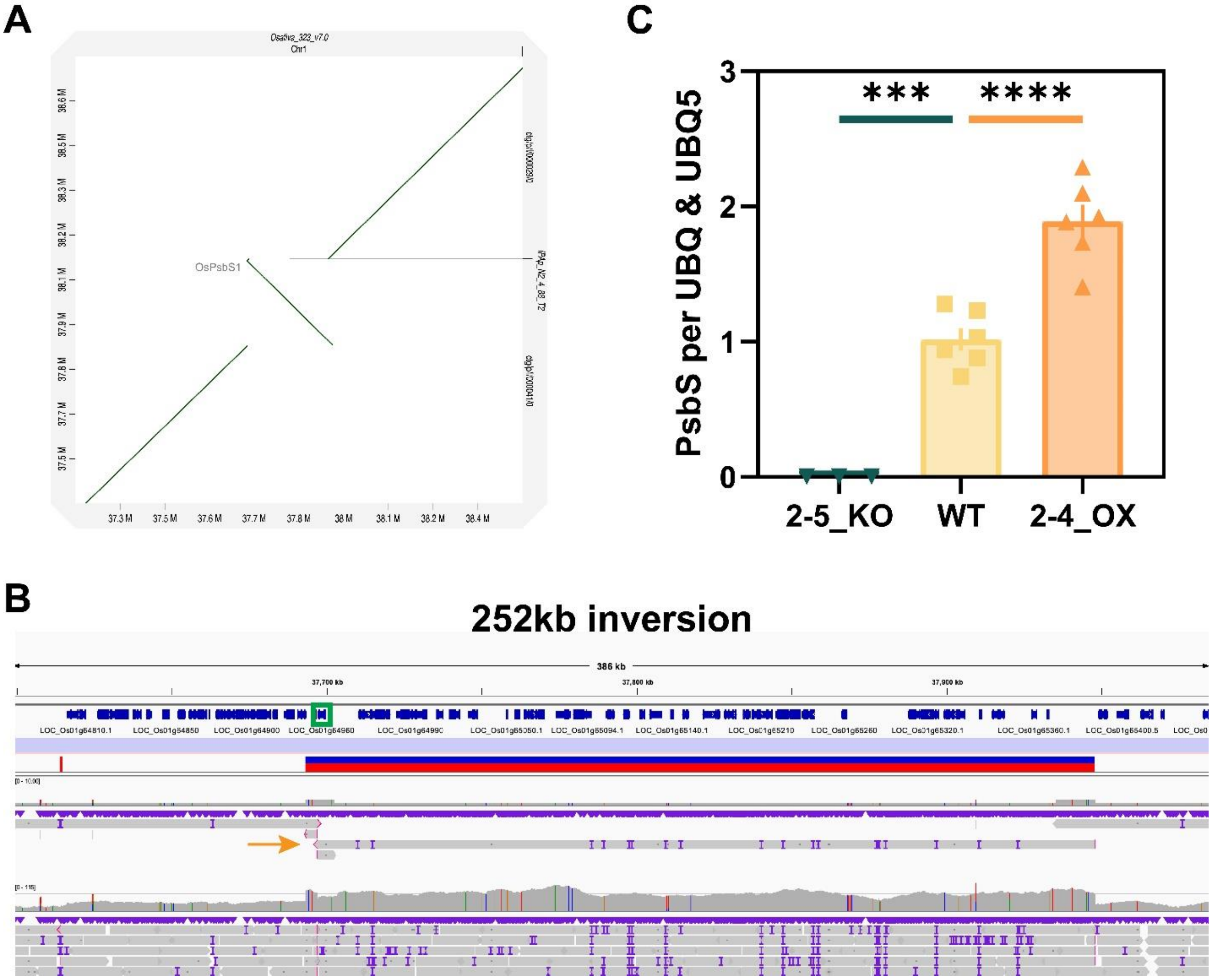

Fig. S6. High resolution view of the 2-4_OX duplication/inversion.

(**A**) Increased resolution of the inversion on the Chr 1 locus (~37.85 Mbp). (**B**) Integrative Genomics Viewer (IGV) display of raw mapped reads. The green box denotes the *OsPSBS1* gene (LOC_Os01g64960). Structural variant called by Sniffles is shown by the red and blue bar. Evidence of the inversion is substantiated by head-to-head sequenced fragments (orange arrow). (**C**) Quantitative PCR analysis to determine PsbS genomic copy number relative to WT and the 2-5 *OsPSBS1* deletion line. Pairwise significance in (C) was determined by ordinary one-way ANOVA (α=0.05) using Dunnett's test for multiple comparisons against Nipponbare WT and is denoted by asterisks (***$p \leq 0.001$, ****$p < 0.0001$).

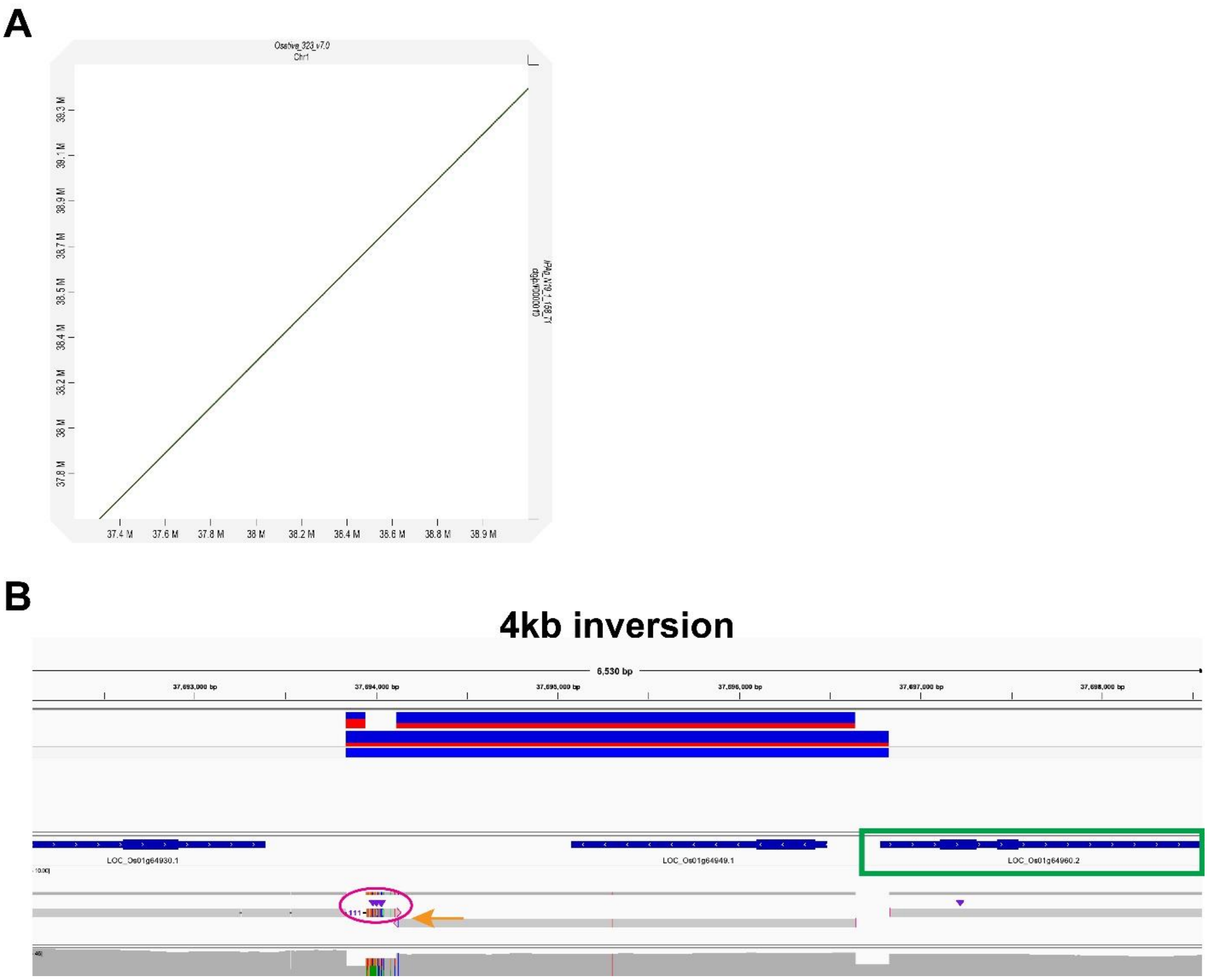

Fig. S7. High resolution view of the 19-1_OX inversion.

(**A**) Increased resolution of the Chr 1 locus (~37.85 Mbp). (**B**) Integrative Genomics Viewer (IGV) display of raw mapped reads. The green box denotes the *OsPSBS1* gene (LOC_Os01g64960). Structural variant called by Sniffles is shown by the red and blue bar (Strength: 0.361). Evidence of the inversion is substantiated by head-to-head sequenced fragments (orange arrow). A non-reference, repetitive element is circled in magenta.

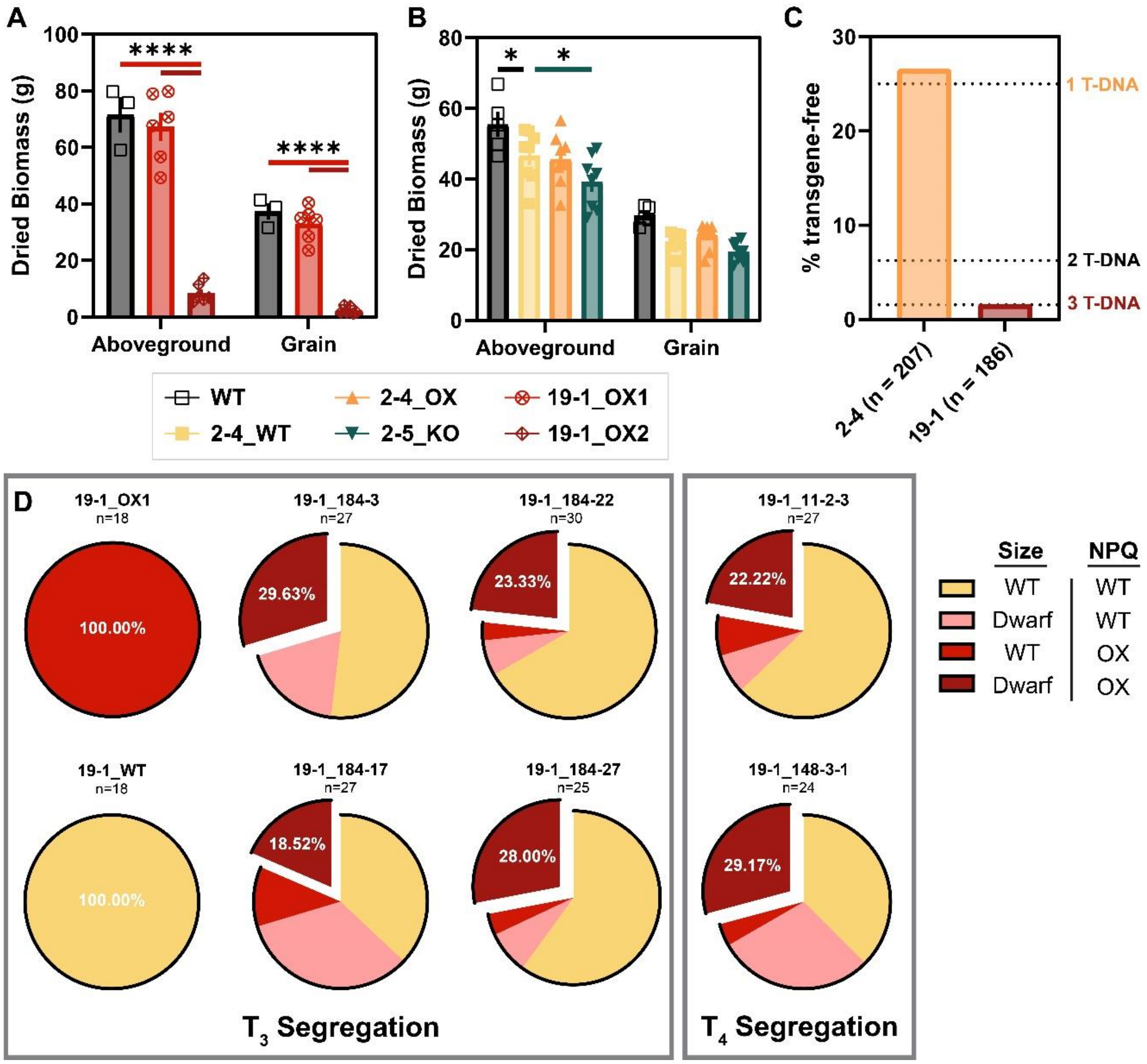

Fig. S8. Compromised biomass and non-mendelian inheritance of 19-1_OX2

Differences in dried aboveground and grain biomass for selected (**A**) Event 19-1 and (**B**) Event 2 alleles. Genotypes are shown: WT (black, open square), 19-1_OX1 (red, crossed circle), 19-1_OX2 (maroon, crossed diamond), 2-5_KO (dark teal, inverse triangle), 2-4_WT (yellow, square), and 2-4_OX (orange, triangle). Pairwise significance in (A,B) was determined by ordinary one-way ANOVA (α=0.05) using Dunnett's test for multiple comparisons against Nipponbare WT or 2-4_WT, denoted by asterisks (*p≤0.05, ****p<0.0001). (**C**) Proportion of T_1 progeny sensitive to hygromycin and lacking the Cas9 transgene. (**D**) Percent inheritance of the parent allele as determined by biomass and NPQ phenotype across several generations. Alleles corresponding to 19-1_WT, 19-1_OX1, and 19-1_OX2 are shown in yellow, red, and maroon, respectively. Progeny dwarfed in size with WT NPQ are shown in pink.

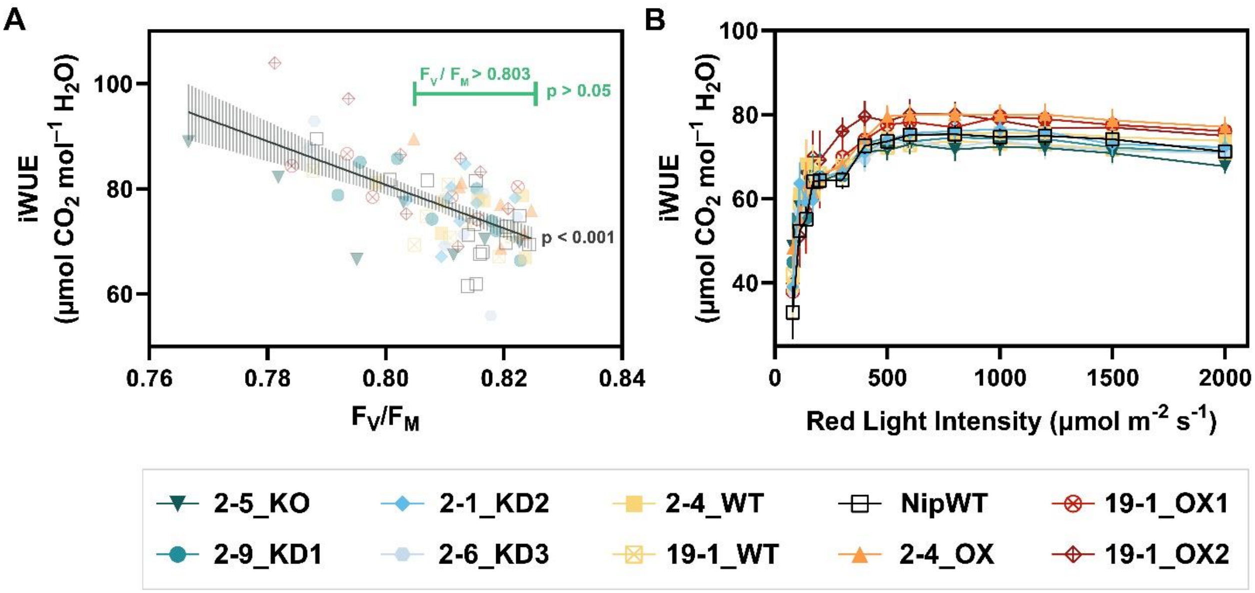

Fig. S9. Correlation of F_v/F_m and iWUE across all genotypes.

(**A**) Linear regression and 95% confidence interval of the average iWUE (µmol CO_2 mol^{-1} H_2O) across all genotypes at light intensities ≥ 500 µmol m^{-2} s^{-1}. Green bars mark the phenotypic range where the genotype-independent correlation between F_v/F_m and iWUE is no longer significantly non-linear. (**B**) iWUE as a function of incident red light on mature flag leaves after constraining analysis to replicates $F_v/F_m > 0.803$ (n=4-7 biological replicates, data shown ± SEM). Genotypes are shown: WT (black, open square), 2-5_KO (dark teal, inverse triangle), 2-9_KD1 (teal, circle), 2-1_KD2 (blue, diamond), 2-6_KD3 (light blue, hexagon), 2-4_WT (yellow, square), 19-1_WT (yellow, crossed square), 2-4_OX (orange, triangle), 19-1_OX1 (red, crossed circle), 19-1_OX2 (maroon, crossed diamond).

Table S1. *OsPSBS1* non-coding sequence gRNA spacers relative to the start codon.

Insert	Orientation (rel. to ORF)	Position from ATG in *O. sativa sp. indica* cultivar IR64	Position from ATG in *O. sativa sp. japonica* cultivar Nipponbare	Spacer Sequence (5' -> 3')
gRNA1	R	-1163 : -1183	-3837 : -3857	GCGAGACACTAAAATACATT
gRNA2	F	-973 : -953	-3647 : -3627	TCTTGTTCCTGGATGTAATT
gRNA3	R	-888 : -908	-3562 : -3582	AGATTCAGGAGTAACAAAAA
gRNA4	F	-769 : -749	-3443 : -3423	TGTTGCATGTGGTCCGTCGA
gRNA5	R	-585 : -605	-3259 : -3279	CACAAAAAAGTACGGGAATG
gRNA6	F	-374 : -394	-374 : -394	TACCAACCACCTGCTCTTCT
gRNA7	R	-263 : -283	-263 : -283	GTCTCCCCGAATCCCTTCTA
gRNA8	F	-166 : -146	-166 : -146	CTACGCCTCCCACCCGCCAC
gRNA scaffold	GTTTTAGAGCTAGAAATAGCAAGTTAAAATAAGGCTAGTCCGTTATCAACTTGAAAAAGTGGCACCGAGTCGGTGC			
tRNA linker	AACAAAGCACCAGTGGTCTAGTGGTAGAATAGTACCCTGCCACGGTACAGACCCGGGTTCGATTCCCGGCTGGTGCA			

Table S2. qRT-PCR primer pairs and observed primer efficiency in 1:81 WT dilution series.

Gene ID	Primer Name	Sequence	Primer Efficiency
UBQ **(67)** LOC_Os03g13170	oDP578q UBQ-F_Fu2020	TGCACCCTAGGGCTGTCAAC	95.3%
	oDP579q UBQ-R-Fu2020	GGCGAGTGACGCTCTAGTTCTT	
UBQ5 **(68)** LOC_Os01g22490	oDP580q UBQ5-F_Jain2006	ACCACTTCGACCGCCACTACT	105.1%
	oDP581q UBQ5-R_Jain2006	ACGCCTAAGCCTGCTGGTT	
PSBS1 **(67)** LOC_Os01g64960	oDP584q PsbS1F-Fu2020	CTGTTCGGCAGGTCCAAG	95.9%
	oDP586q PsbS1R2-DP	CAAACCCGAGCATGGCGA	

Table S3. Primers for PCR amplification and genotyping of *OsPSBS1* non-coding sequences.

Amplicon	Forward Primer (5' -> 3')	Reverse Primer (5' -> 3')	Amplicon Size	Additional Sequencing Primer
Distal end (gRNA 1-5)	oDP214: AGACAGAGGTATGTCAATGTGTTATTG	oDP498: AAAGAGCAAATGGCCTACCA	2384bp	oDP500: CGTGTCTCCCACGTCTTCTT
Proximal end (gRNA 6-8)	oDP497: TGGTAGGCCATTTGCTCTTT	oDP496: CATCCATCCAAATTCCAACCT	2167bp	oDP499: GAGCAAACACTCAGGCACAA
